# Single cell transcriptomic analysis of human mesenchymal stem cells reveals limited heterogeneity

**DOI:** 10.1038/s41419-019-1583-4

**Published:** 2019-05-08

**Authors:** Yin Huang, Qing Li, Kunshan Zhang, Mingyuan Hu, Yu Wang, Liming Du, Liangyu Lin, Siguang Li, Lydia Sorokin, Gerry Melino, Yufang Shi, Ying Wang

**Affiliations:** 1 CAS Key Laboratory of Tissue Microenvironment and Tumor, Shanghai Institute of Nutrition and Health, Shanghai Institutes for Biological Sciences, University of Chinese Academy of Sciences, Chinese Academy of Sciences, 200031 Shanghai, China; 2 Translational Stem Cell Research Center, Tongji Hospital, Tongji University School of Medicine, 200065 Shanghai, China; 3 Institute of Physiological Chemistry and Pathobiochemistry, University of Münster, Münster, Germany; 4 Biochemistry Laboratory IDI-IRCC, Department of Experimental Medicine and Surgery, University of Rome Torvergata, 00133 Rome, Italy; 5 Soochow University Institutes for Translational Medicine, Soochow University, Suzhou, China

**Keywords:** RNA sequencing, Mesenchymal stem cells

## Abstract

Mesenchymal stem cells (MSCs) are a population of multipotent cells with a superior ability to promote tissue repair by regulating regeneration and inflammation. Effective application of MSCs in disease treatment relies on the production of relatively homogeneous cell population. However, the cellular heterogeneity and the differentiation trajectories of in vitro expanded MSCs remain largely unclear. We profiled the transcriptomes of 361 single MSCs derived from two umbilical cords (UC-MSCs). These UC-MSCs were harvested at different passages and stimulated with or without inflammatory cytokines. Weighted gene correlation network analysis revealed that UC-MSCs surprisingly possess only limited heterogeneity, regardless of donors, and passages. We also found that upon pretreatment with inflammatory cytokines (IFNγ and TNFα), a classical strategy that can improve the efficiency of MSC-based therapy, MSCs exhibited uniformed changes in gene expression. Cell cycle-based principal component analysis showed that the limited heterogeneity identified in these UC-MSCs was strongly associated with their entrance into the G2/M phase. This was further proven by the observation that one featured gene, CD168, was expressed in a cell cycle-dependent manner. When CD168^high^ UC-MSCs were sorted and cultured in vitro, they again showed similar CD168 expression patterns. Our results demonstrated that in vitro expanded UC-MSCs are a well-organized population with limited heterogeneity dominated by cell cycle status. Thus, our studies provided information for standardization of MSCs for disease treatment.

## Introduction

Mesenchymal stem cells (MSCs) are multipotent cells that were discovered from bone marrow (BM) by a Russian scientist, Alexander Friedenstein, in the late 1960s^1^, following his observations that BM is a stem cell reservoir for mesenchymal tissues in postnatal life. Nowadays, the identification of MSCs is based on their fibroblast-like morphology, the expression status of a panel of surface markers, and their trilineage differentiation potentials^2^. Due to their self-renewal property and the multiple differentiation potential, therapies using in vitro expanded MSCs in regenerative medicine are highly promising.

MSCs have been isolated from various tissues and organs. Although bone marrow derived MSCs (BM-MSCs) are most widely used for therapeutic potential explorations, umbilical cords (UC)-MSCs seem to be better-suited candidates for clinical applications. Compared to BM-MSCs, UC-MSCs are available in large quantities and can be easily harvested, devoid of the troublesome BM aspiration procedures. Importantly, UC-MSCs are derived at an early stage of life and possess lower immunogenicity when transplanted in vivo. Although they are mostly used allogeneically, their therapeutic efficacy has been demonstrated in several preclinical and clinical studies^3,4^.

In the past decades, investigations on MSCs in tissue regeneration have achieved impressive successes in managing otherwise uncontrollable inflammatory diseases, a capability attributed to the immunoregulatory capabilities of MSCs. The astonishing efficacy of MSCs in treating inflammatory diseases was first found in a 9-year-old patient suffering from steroid and cyclosporine resistant GvHD^5^; however, successful outcomes are not always achievable^6^. Considering the key role of inflammation in enabling MSCs with the immunosuppression ability, a series of investigations using a combination of inflammatory cytokines, IFNγ and TNFα, to pretreat MSCs in vitro have attained improved therapeutic effects on autoimmune disorders in vivo^7,8^. Robust expression of chemokines and immunosuppressive factors in MSCs, such as CCL5, CXCL9, indoleamine 2,3-dioxygenase (IDO), prostaglandin-endoperoxide synthase 2 (PTGS2), and TNFα-stimulated gene-6 (TSG6) can be induced by inflammatory cytokines to lead to immunosuppression by a concerted action^9^.

Among the prominent parameters controlling the efficacy of MSCs in clinical applications, the standardization of MSC populations is on the top of the list. It has been reported that in vitro expanded MSCs were diverse in morphology and proliferation rates^10^. In addition, MSCs derived from different clones showed different colony formation and differentiation abilities^11^. These evidences indicate that heterogeneities could exist in MSCs derived from different donors, tissues, clones and even different cells from the same clone^12^. Pioneer work in a murine model of delayed type hypersensitivity (DTH) revealed that MSCs isolated from different donors showed distinct effects on treating DTH^13^. Thus, inter-clonal heterogeneity is a possible explanation for the inconsistent results in clinical trials. Advances in single cell sequencing provide a unique approach to explore the heterogeneity at the single cell level. Up to date, most studies on human MSCs are based on bulk populations, detailed investigations on single cell analysis of MSCs to elucidate the variations in various properties are urgently needed.

Here, we employed a state-of-the-art microfluidic single cell capture system and next-generation sequencing technology to investigate the transcriptomic heterogeneity of human UC-MSCs. We profiled 361 UC-MSCs, and 159 of them were exposed to inflammatory cytokines. We revealed that there generally existed subpopulations of cultured UC-MSCs. Through weighted gene correlation network analysis (WGCNA)^14,15^, we found the heterogeneities of UC-MSCs are dominated by certain key gene modules. Further analysis based on these genes proved that cell cycle distribution is an important reason for the sources of heterogeneity in UC-MSCs. Combined with information from other studies, our results indicated that UC-MSCs are a comparatively homogeneous cell population and standardization in isolation and expansion protocols is highly possible for achieving better clinical results.

## Results

### Heterogeneity in human umbilical cord derived MSCs

**Fig. 1:**
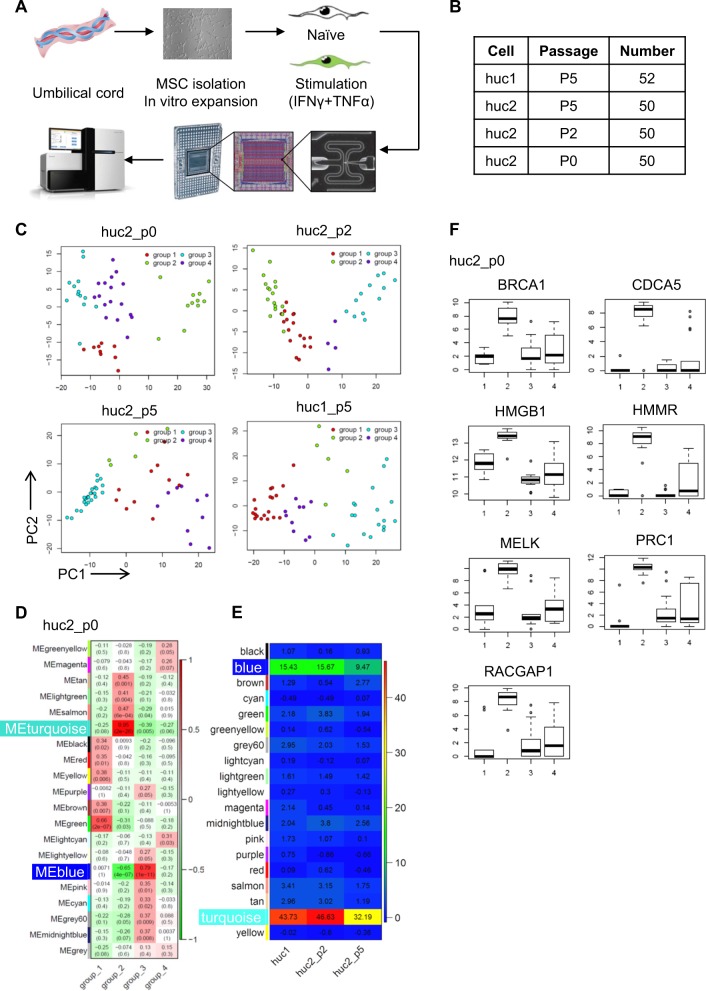
Heterogeneity in human umbilical cord derived MSCs. **a** Process for preparation of MSCs for single cell sequencing, including MSCs isolation from the umbilical cord, in vitro expansion, pretreatment approach, single cell capture, and RNA sequencing. **b** Sample information for RNA sequencing. **c** PCA analysis performed on UC-MSCs from the two different donors and three passages. Each plot represents an independent analysis. Cells are grouped into four distinct clusters. **d** Key modules of MSC heatmap revealed by WGCNA, with the significance of each module in each subcluster. Each cell contains the *p* value. Data are representative from huc2_p0. **e** Heatmap of module preservation scores among different datasets. Module preservation scores are represented by the *Z*-summary score. **f** Expression profile of featured genes in four subclusters are shown as box plot. Data are representative from huc2_p0

Single cell transcriptome analysis has been widely used to identify cellular signature and to reveal developmental trajectory. To examine the heterogeneity features of MSCs and add in their clinical applications, we isolated MSCs from human umbilical cords, expanded in vitro, and performed single cell RNA sequencing (Fig. 1a). These in vitro expanded UC-MSCs met the requirements of MSCs definition, such as cell surface positive for CD73, CD90, CD105, and negative for CD31, CD34, CD45, and HLA-DR (Fig. S1).

We isolated individual UC-MSC (52 cells from donor 1 in passage 5, 50 cells from donor 2 in passage 0, 50 cells from donor 2 in passage 2, 50 cells from donor 2 in passage 5) (Fig. 1b) by the Fluidigm C1 Auto-prep system and 96 × 96 integrated fluidic circuits. After RNA extraction, cDNA preamplification and sequencing quality control, we prepared cDNA library of all 361 cells (159 cells with and 202 cells without stimulation), and sequenced the transcriptome of these cells on an Illumina HiSeq 2500 system. High uniformity in a high sensitivity DNA assay was verified (Fig. S2).

By analyzing the single cell RNA-sequencing data of 202 untreated cells using spike-in RNA 1,4,7 or ERCC, and reference genes as inner control, we identified highly variable genes, and found several subclusters among all four datasets (huc2_p0, huc2_p2, huc2_p5, huc1_p5) through principal component analysis (PCA) (Fig. 1c). Among the genes examined, 4753 out of 5498 genes passed the filter of low-expression genes (detected in at least 1/10 cells and average 1 reads per cell), and were applied to WGCNA of each dataset. This result revealed that in primary UC-MSCs, the blue module (0.79, *p* = 1 × 10^−11^ in huc2_p0) and the turquoise module (0.95, *p* = 2 × 10^−26^ in huc2_p0) were significantly correlated with one of the subpopulations (group 3 and 2 respectively in huc2_p0) (Fig. 1d). A similar pattern was observed in four datasets (Fig. S3). The shared genes in the blue and turquoise modules in four datasets were shown in Venn plot (Fig. S4). Z-summary score also indicated that both of them are highly conserved (blue: huc1_p5: 15.43, huc2_p2: 15.67, huc2_p5: 9.47; turquoise: huc1: 43.73, huc2_p2: 46.63, huc2_p5: 32.19) (Fig. 1e). These results demonstrated that the existence of subpopulations in human UC-MSCs, and these subpopulations were conserved independent of donors and passages.

In order to find characteristic markers for different subpopulations of MSCs, we screened the genes in blue and turquoise modules for those highly expressed in one specific group and existed in more than one dataset (Fig. S5, group 3 in dataset huc1, group 2 in dataset huc2_P0, group 3 in dataset huc2_P2, and group 4 in dataset huc2_P5). Subsequently, we identified seven featured genes: *brca1*, *cdca5*, *hmgb1*, *hmmr/cd168*, *melk*, *prc1*, and *racgap1*. Their expression patterns in all four subclusters are similar and were shown in huc2_p0 as representative (Fig. 1f and Fig. S5). All seven genes were significantly higher in group 2 as compared to other groups. Collectively, we found UC-MSCs possess heterogeneity in the expression of some genes.

### Inflammatory cytokines stimulated MSCs subpopulations also exhibit heterogeneity in the featured genes

**Fig. 2:**
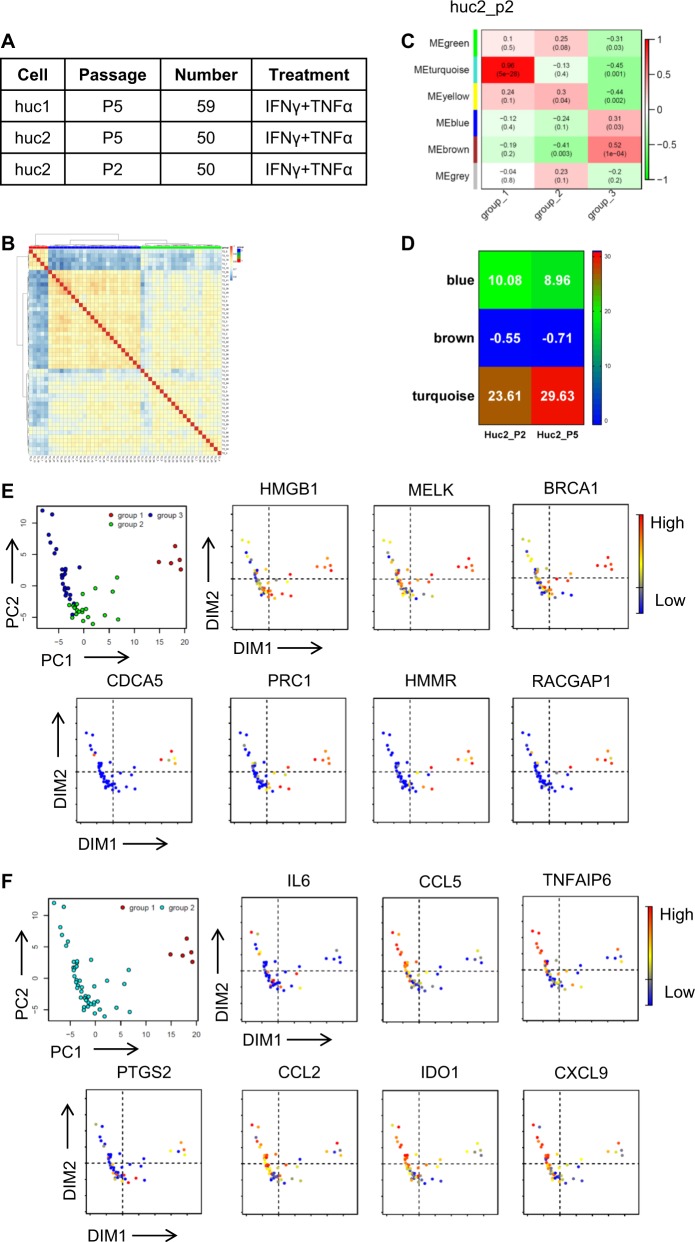
Inflammatory cytokines stimulated MSCs subpopulations also exhibit heterogeneity in the featured genes. **a** Sample information for RNA sequencing. Cells are pretreated with IFNγ and TNFα (10 ng/mL) for 12 h. **b** Unsupervised hierarchical clustering of MSCs with IFNγ and TNFα pretreatment. Data are representative from huc2_sti_p2. **c** WGCNA reveals the key modules of the heatmap of MSCs stimulated by inflammatory cytokines, IFNγ and TNFα, showing the significance of each module in each subcluster. Each cell contains the *P* value. Data are representative from huc2_sti_p2. **d** Heatmap of module preservation score among different datasets. Module preservation scores are represented by the *Z*-summary score. **e, f** PCA analysis shows the gene clustering in huc2_sti_p2 as representative. Expression profile of featured genes (**e**) and immunomodulatory genes (**f**) is analyzed. Red to blue shows the level from high to low

One of the most inimitable properties of MSCs is their immunosuppressive function and the effectiveness in treating various inflammatory diseases, such as GvHD, inflammatory bowel disease, and multiple sclerosis^16^. Such immunosuppressive ability of MSCs is found to be endowed by inflammation^17^. In the presence of inflammatory cytokines, IFNγ and TNFα, human MSCs exhibit exuberant expression of IDO, TSG6, PTGS2/COX2, various chemokines, and many growth factors^18–20^. Consequently, several studies have shown that inflammatory cytokines pretreated MSCs exhibited better therapeutic effects^7,8,13,21–23^. The question now is whether there is heterogeneity in inflammation-induced gene expression in MSCs. Upon stimulation with IFNγ and TNFα, 159 cells from UC-MSCs (huc1_sti_p5, huc2_sti_p2, and huc2_sti_p5) were analyzed (Figs 1a and 2a). Through unbiased hierarchical clustering, one distinguished subpopulation could be recognized in all datasets (Fig. 2b and Fig. S6). By using WGCNA, the turquoise and blue modules were found to be significantly correlated and conserved in different datasets. Additionally, genes in these two modules were largely similar to those identified in untreated UC-MSCs (Fig. 2c, d and Fig. S7). Interestingly, after the treatment with inflammatory cytokine, the dominant modules of MSCs still showed similar pattern as unstimulated state.

To ascertain whether the featured genes in naïve UC-MSCs would also distinguish subpopulations in inflammatory cytokines primed MSCs, we performed PCA. We found that the expression levels of seven featured genes found previously were the highest in group 1 among the 3 groups (Fig. 2e). Intriguingly, the expression of key cytokines and chemokines for MSCs-mediated immune modulation (including CCL5, IDO1, CXCL9, CCL2, PTGS2/COX2, TNFAIP6/TSG6, and IL6) was the lowest in group 1 compared to group 2 and 3, (Fig. 2f), while other genes exhibited a similar expression pattern in all 3 groups. These inverse correlations of the featured genes and immune modulatory genes could be found in all datasets (Figs. S8 and S9). These results demonstrated that the subpopulations we found in UC-MSCs express high levels of the seven featured genes while express low levels of immunomodulatory genes.

### The limited heterogeneity in expanded MSCs is linked to cell cycle stages

**Fig. 3:**
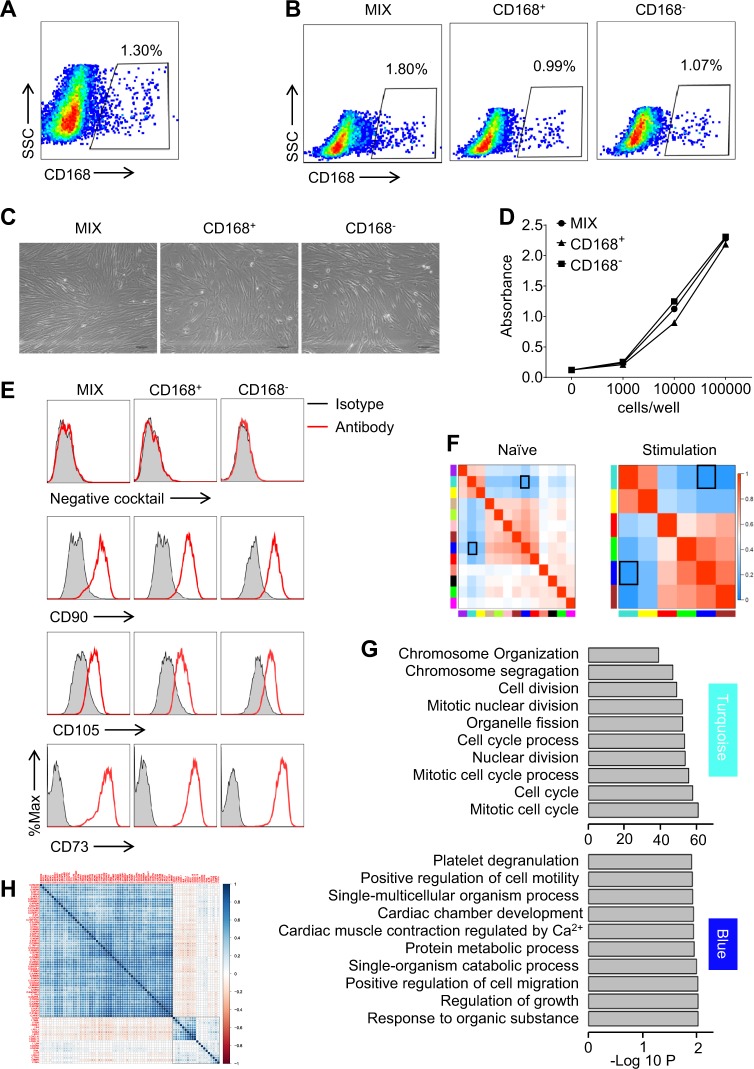
The limited heterogeneity in expanded MSCs is linked to cell cycle stages. **a** CD168^+^ cells are sorted from in vitro expanded MSCs by flow cytometry. **b** CD168^+^ cells and CD168^−^ cells are sorted from in vitro expanded MSCs. After in vitro expansion, these cells, together with in vitro expanded MSCs (MIX), were analyzed for the frequency of CD168^+^ cells by flow cytometry. **c** CD168^+^, CD168^−^, and MIX cells shared similar spindle-shaped morphology. **d** Proliferation rate is shown as absorbance at 490 nm after the addition of MTS for 4 h. *X-*axis represents cell number seeded in 96-well plate (cells/well). **e** MSCs marker analysis by flow cytometry. Negative cocktail included CD34, CD11b, CD19, CD45, and HLA-DR. Red line is designated antibody and gray area is the isotype control. **f** Correlation analysis among modules is revealed by WGCNA. Data are representative from huc1. Black square shows the reverse correlation of blue and turquoise module. **g** GO analysis of genes is performed in turquoise and blue modules. **h** 77 genes in MSCs are detected by single cell qPCR. Heatmap shows the correlation between immune regulation-related genes and cell cycle-related genes. Red to blue represents low to high expression level of designated genes

Given that the featured genes are representatives of the variability of gene expression among individual cells, we asked whether these featured genes can be employed to identify subpopulations of MSCs. We evaluated the cell population with featured gene expression in expanded UC-MSCs. Only a small population of UC-MSCs can be observed to express high levels of featured genes (Fig. S10). Among these featured genes, CD168 showed a relatively higher expression level than other genes. We then isolated CD168^+^ MSCs and CD168^−^ MSCs by flow cytometric sorting (Fig. 3a). Together with unsorted MSCs, we compared their morphology, cell surface markers, and proliferation potentials. Microscopically, these cells exhibited similar spindle-shaped morphology (Fig. 3c). By employing the MTS assay to determine their proliferation potential, we found that the growth rates of CD168^+^ MSCs, CD168^−^ MSCs and unsorted MSCs are similar, regardless of the amount of cells seeded (Fig. 3d). Also, we evaluated a panel of markers for MSC identification among the three groups, including CD73, CD90, CD105, CD34, CD11b, CD19, CD45, and HLA-DR. No significant difference was observed for positive expression of CD73, CD90, CD105, and negative expression of CD34, CD11b, CD19, CD45, and HLA-DR (Fig. 3e). Intriguingly, when we analyzed the expression of CD168 in expanded CD168^+^ MSCs, CD168^−^ MSCs, and unsorted MSCs, we found that the CD168 expression levels among these groups were comparable (0.99%, 1.07%, and 1.80%, respectively) (Fig. 3b). These results suggested that CD168^−^ MSCs can regain the gene expression patterns as CD168^+^ MSCs did upon expansion, indicating a limited heterogeneity among MSCs.

To investigate the characteristics and formation of the limited heterogeneity among MSCs, we analyzed the relationship among various modules in each dataset, as discribed in Fig. 3f. Of note, we have shown above that among the blue and the turquoise gene module, there were significant variabilities of gene expression in UC-MSCs with or without inflammatory stimulation (−0.69 and −0.88 in huc1_ctrl and huc1_sti, respectively) (Fig. 3f). Gene ontology (GO) term enrichment analysis further showed that the terms related to cell migration and cytokine signaling were representative in the blue module, and the terms related with cell cycle activity were highly enriched in the turquoise module (Fig. 3g).

We further compiled a list of 57 cell cycle-associated genes and 20 immunoregulation-related genes based on the annotations in the GO term “cell cycle” and “immunoregulation”, and performed single cell qPCR to determine their expression in expanded UC-MSCs. Consistently, we found a highly reverse correlation within cell cycle-related genes and immune modulation-related genes between the two big blocks (Fig. 3h). Such reverse correlation can also be observed in single cell RNA sequencing of UC-MSCs (Fig. 2b, c and Figs. S8 and 9). Thus, our results demonstrated that the limited heterogeneity in UC-MSCs is related with cell cycle progression.

### Cell cycle stage determines the heterogeneity of MSCs

**Fig. 4:**
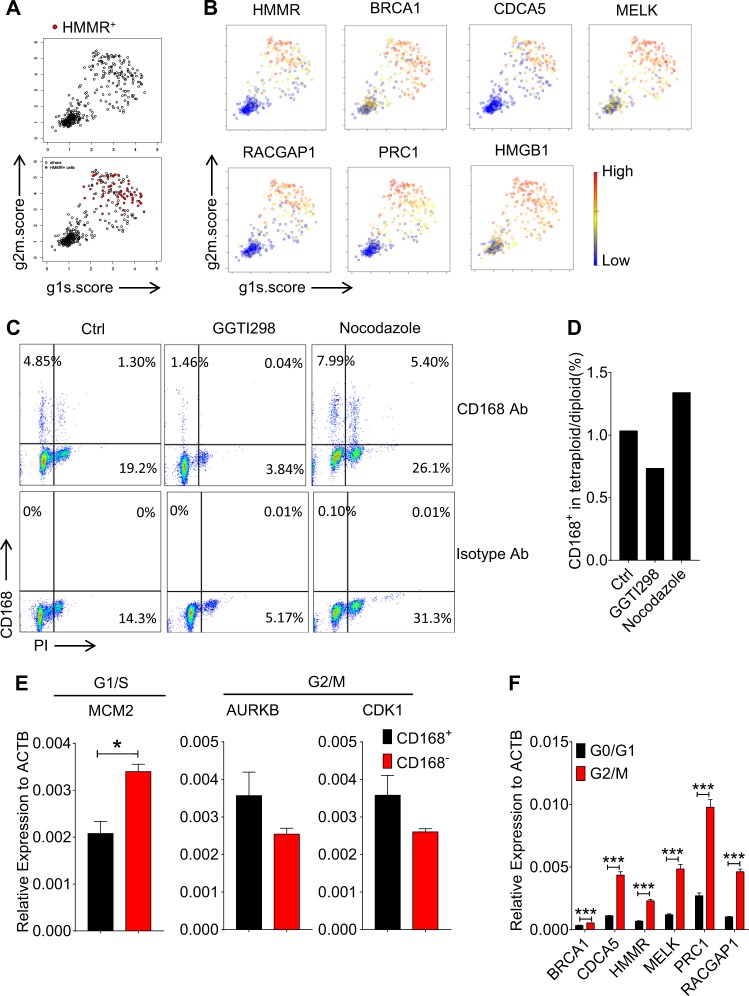
Cell cycle stage determines the heterogeneity of MSCs. **a** Classification of cells to noncycling (bottom left) and cycling cells (top right) based on the relative expression of gene-sets associated with the G1/S stage (*x* axis) and the G2/M stage (y axis). CD168/HMMR^+^ cells are labeled as red dots. **b** Hierarchy plot for the featured genes, with each cell color-coded based on the expression level. Red denotes high and blue is representative for low. **c**, **d** Flow cytometry analysis (**c**) and bar plot (**d**) show the CD168 expression and cell cycle distribution on MSCs with or without GGTI298 (2.5 μM), or nocodazole (1 μg/ml) treatment for 24 h. **e**, **f** CD168^+^ MSCs are sorted by flow cytometry. Cell cycle-related genes are analyzed (**e**). MSCs in different cell cycle stages are sorted by Hoechst staining. Featured genes, BRCA1, CDCA5, HMMR, MELK, PRC1, and RACGAP1, are analyzed by real-time PCR. Black bars and red bars represent cells in G0/G1 stage, and cells in G2/M stage respectively (**f**). In this figure, data are represented as Mean ± SEM. *p < 0.05; **p < 0.01; ***p < 0.001. *ns* no significance; by unpaired two-tailed Student’s *t*-test

We next examined the influence of cell cycle trajectory on the appearance of heterogeneity in UC-MSCs. We employed the classification methods based on scoring cell cycle-related genes and plotted each cell for the expression of signature genes of cells in the G1/S and G2/M phases^24^. By showing the CD168/HMMR^+^ cells in red, we found that most of the cells were on the top right, indicating that they are in the G2/M phase (Fig. 4a). To further confirm the correlation of other featured genes (*brca1*, *cdca5*, *melk*, *racgap1*, *prc1*, and *hmgb1*) related to cell cycle, we checked their expression patterns, and found their expression kinetics were similar to CD168/HMMR^+^ MSCs, enriched on the top right of the plot (Fig. 4b). To examine whether the cell cycle stages are related to the high expression of these signature genes in UC-MSCs, we employed four small molecule inhibitors to arrest MSCs at the G1/S phase (Abemaciclib and GGTI298) and the G2/M phase (Nocodazole and Ixabepilone) (Fig. 4c and Fig. S11). With propidium iodide (PI) staining to show the DNA content in MSCs and to ascertain the phases of cell cycle, we analyzed the percentages of CD168^+^ cells in diploid and tetraploid cells. We found that most MSCs arrested in G2/M phase were positive for CD168 (Fig. 4c, d and Fig. S11). However, UC-MSCs arrested in the G1/S phase exhibited an opposite pattern (Fig. 4c, d and Fig. S11).

Since in vitro culture highly reduced the expression of CD168/HMMR in UC-MSCs, we analyzed the cell cycle-related gene expression in isolated CD168/HMMR^+^ MSCs directly. As predicted, freshly isolated CD168/HMMR^+^ MSCs expressed lower levels of G1/S gene, *mcm2*, while exhibited higher expression of G2/M gene, *aurkb*, and *cdk1* (Fig. 4e). We thus illustrated the relationship of the G2/M phase of the cell cycle with these indicated featured genes, including *brca1*, *cdca5*, *hmmr*, *melk*, *prc1*, and *racgap1*. We sorted UC-MSCs in G0/G1 phase and G2/M phase, and analyzed the expression of the forementioned genes using real-time PCR. We found that these featured genes are expressed highly in the G2/M cells than that in the G0/G1 cells (Fig. 4f). Taken together, cell cycle stage controls the heterogeneity of UC-MSCs.

## Discussion

Since the first case report of MSC application in a 9-year-old boy suffering from steroid and cyclosporine resistant GvHD, various follow-up studies focused on their paramount potential in tissue repair and regeneration^16,25^. Their successful application is relied on several key parameters, including the quality of MSCs, injection route, patient status, and clinical interventions^16^. Among them, the standardization of MSCs is foremost important to control the quality of MSCs. Here, we performed single cell RNA sequencing on UC-MSCs from two different donors and three passages, with or without inflammatory cytokine stimulation. We found that the heterogeneity of gene expression among UC-MSCs is limited, regardless of the donors, and passages. Also, UC-MSCs stimulated by inflammatory cytokines, IFNγ and TNFα, showed similar diversity patterns in gene expression, indicating limited heterogeneity of MSCs with and without inflammatory cytokine stimulation. We found seven featured genes including *brca1*, *cdca5*, *melk*, *hmmr*, *racgap1*, *prc1*, and *hmgb1*. Further analysis revealed that to a large extent, the heterogeneity is related to cell cycle stages of UC-MSCs. Pharmacological arrest of MSCs at G0/G1 phase or G2/M phase can regulate the expression of these featured genes. Thus, cell cycle trajectory leads heterogeneity among UC-MSCs.

After their initial identification from bone marrow, MSCs now can be obtained from almost all tissues^26–28^. Although BM-MSCs are the most popular cells in clinical trials, compared to MSCs derived from other tissues, UC-MSCs possess great potential for large-scale expansion and for standardization^27^. Single cell gene profiling on UC-MSCs showed heterogeneity in individual cell, and different cells exhibited distinct proliferation and differentiation potentials^29^. Classification of UC-MSCs based on their sizes revealed that smaller cells grow faster and age slower than larger cells^30^. Also, employment of leveraging multicolor lentiviral barcode labeling as a tool for UC-MSC clonal development analysis revealed that the heterogeneity in MSCs can decrease during their in vitro expansion^31^. Therefore, recognition and manipulation of heterogeneity among in vitro expanded MSCs can represent novel therapeutic opportunities for their application in disease treatment. Considering different donor sources and the possibility of clonal selection of MSCs during the expansion process, our single cell RNA-sequencing analysis on UC-MSCs from different donors and passages delineated that the variability among the datasets is much conserved, indicating limited heterogeneity of UC-MSCs. Previous studies have pointed out that culture condition can also influence the transcriptional and proteome profile of MSCs^32^. In the presence of inflammation, MSCs from distinct donors can provide the consistent results in resolving the inflammation of DTH, attributing to comparable and abundant expression of immunosuppressive molecules in distinct donor MSCs initiated by inflammatory stimuli. These results raise a potential approach to develop heterogeneous MSCs into homogeneous populations. Although inflammation can license MSCs with paramount immunosuppression and enable distinct donor-derived MSCs with similar improvement in tissue regeneration, these inflammatory cytokine-stimulated UC-MSCs are not homogenous. Surprisingly, their expression pattern for a variability of gene profile was similar to that of untreated UC-MSCs.

In order to figure out the essence of limited heterogeneity of UC-MSCs, we analyzed the featured genes, and found the turquoise module has a very close relationship with cell cycle regulation. A reverse correlation between the module clustered with cell cycle gene and the module clustered with immune related molecules was surprisingly observed. Such relationship between inflammatory cytokine and cell cycle gene expression has been reported in colon cancer cells^33–35^. Similar phenomenon can be observed in senescence-associated secretory phenotype. Senescent cells undergo cell cycle arrest while acquire the ability to promote inflammation^36^, further supporting an inverse correlation between cell cycle and immune regulatory property of MSCs. By utilizing the cell cycle scoring algorithm recently developed^24^, we found the appearance of subcluster in MSCs was mostly in G2/M phase, and the major heterogeneity of UC-MSCs, with or without inflammatory cytokines, is controlled by cell cycle progression.

In conclusion, our analysis of UC-MSCs through single cell RNA sequencing revealed that the heterogeneity of UC-MSCs is mainly caused by distinct distribution in cell cycle phases. In addition to be valuable in studying MSC biology, this information has critical implications for MSC standardization and further development of strategies using MSCs to treat various diseases.

## Methods and materials

### Cell isolation and expansion

Umbilical cord was harvested from fetuses with natural labor from Changzhou Maternal and Child Health Care Hospital, temporally preserved in saline with the addition of 100 U/mL penicillin, 100 U/mL streptomycin, and 0.54% sodium citrate anticoagulant. Cells were isolated as described below. Umbilical cord was washed with saline supplement with antibiotics. After the blood vessels were removed, the tissue was excised into pieces with diameter around 1 cm. The tissue clumps were cultured in the petri dish with low-glucose DMEM supplemented with 10% fetal bovine serum, 100 U/mL penicillin, and 100 U/mL streptomycin. Half of culture medium was changed on day 4. At day 12, all the clumps were removed and cells were cultured for 1 more week. When cells reached about 80% confluency, they were passaged and cultured under the condition mentioned above. Samples are all qualified for microbiology and virology tests. All individuals were provided with informed consent, and the experiments were approved by the institutional biomedical research ethics committee of the Shanghai Institutes for Biological Sciences (Chinese Academy of Sciences) and Changzhou Maternal and Child Health Care Hospital.

### Single cell capture and quality control

MSCs were captured on a large microfluidic chip (designed for cells from 17 to 25 μm) using the Fluidigm C1 Autoprep System. Capturing efficiency was evaluated by microscopic observation; each capture site was manually confirmed with single cell and processed further. RNA degradation and contamination was monitored on 1% agarose gels. RNA purity was checked using the NanoPhotometer spectrophotometer (IMPLEN, CA, USA). RNA concentration was measured using the Qubit RNA Assay Kit in Qubit 2.0 Flurometer (Life Technologies, CA, USA). RNA integrity was assessed using the RNA Nano 6000 Assay Kit of the Bioanalyzer 2100 system (Agilent Technologies, CA, USA). Quality control was performed by Novogene Co., Limited.

### Library preparation and sequencing

Sequencing libraries were generated using the NEBNext Ultra RNA Library Prep Kit for Illumina (NEB, USA) following manufacturer’s recommendations and index codes were added to attribute sequences to each sample. PCR products were purified (AMPure XP system) and library quality was assessed on the Agilent Bioanalyzer 2100 system. The clustering of the index-coded samples was performed on a cBot Cluster Generation System using the TruSeq PE Cluster Kit v3-cBot-HS (Illumia) according to the manufacturer’s instructions. After cluster generation, the library preparations were sequenced on an Illumina Hiseq 2500 platform and 50 bp single-end reads were generated. These procedures were achieved by Novogene Co., Limited.

### Data analysis

Raw data (raw reads) of fastq format were firstly processed through in-house perl scripts. In this step, clean data (clean reads) were obtained by removing reads containing adapter, poly-N and low quality reads from raw data. At the same time, the clean data of Q20, Q30 and GC content were calculated. All the downstream analyses were based on the clean data with high quality.

Reference genome (hg19) and gene annotation file (UCSC RefGene) were downloaded from illumina iGenomes. Index of the reference genome was built using Bowtie2 (v2.2.5) and single-end clean reads were aligned to the reference genome using Top Hat (v2.1.0).

### Quantification of gene expression level

HTSeq v0.6.1 was used to count the reads numbers mapped to each gene. To normalize sequencing depth, the size factors was calculated by estimate SizeFactorsForMatrix function in R package DESeq2. Normalized counts were calculated by divided raw counts of each genes by size factor of each samples.

### Subgroup identification and differential expression analysis

We applied R package SCDE to identify subpopulations of MSCs. Briefly, spike-in RNAs were used to calculate a regression line of gene expression levels and variations, single-cells were grouped into subpopulations by hierarchical clustering with genes that significantly deviation from the fit. Differential expression analysis of two conditions/groups (two biological replicates per condition) was performed using the SCDE R package. The SCDE package implements routines for fitting individual error models for single-cell RNA-seq measurements by using a mixture of a negative binomial distribution and low-level Poisson distribution to model each gene. Genes with *P*-value < 0.05 found by SCDE were assigned as differentially expressed.

### GO analysis of differentially expressed genes

GO enrichment analysis of differentially expressed genes was implemented by the topGO R package. GO terms with corrected *P* value < 0.05 were considered significantly enriched by differential expressed genes.

### Weighted gene correlation network analysis (WGCNA)

A signed network was constructed by using genes that significantly deviated from SCDE fit in each dataset. Soft power 12, which is the default parameter, was used to derive a pair wise distance matrix for selected genes using the topological overlap measure, and the dynamic hybrid cut method was used to detect clusters. The node centrality, defined as the sum of within-cluster connectivity measures, was used to rank genes for “hub-ness” within each cluster. For visual analysis of the constructed networks by hard thresholding of edge distances, the closest 150 edges were represented using Cytoscape 3.0.0.

Based on the gene modules identified by WGCNA analysis, we screened the genes in blue and turquoise modules with three criteria: (1) highly expressed in one specific subcluster compared to the other clusters; (2) the subcluster specific expression existed in more than one dataset; (3) expressed on the cell surface. Finally, we identified seven featured genes: brca1, cdca5, hmgb1, hmmr/cd168, melk, prc1, and racgap1.

### Flow cytometry

Cells surface markers were detected according to the R&D flow cytometry protocol. Briefly, cells were harvested and washed with PBS. Cells were then resuspended in PBS containing 0.5% bovine serum albumin and were incubated on ice for 30 min with rabbit anti-human CD168 antibodies, followed by another 30 min staining with goat anti-rabbit IgG (H + L) cross-adsorbed secondary antibody-Alexa Fluor 647. The stained cells were washed and analyzed on a FACS Calibur flow cytometer (Becton Dickinson, San Jose, CA, USA). Cell cycle analysis using PI was performed. FlowJo was used to analyze the data.

### Cell proliferation assay

MTS/PMS (3-(4,5-dimethylthiazol-2-yl)-5-(3-carboxymethoxyphenyl)-2-(4-sulfophenyl)-2H-tetrazolium) (Promega, Madison, WI, USA) was used to measure the growth rate of the cells according to the manufacturer’s protocol. Briefly, 20 μl MTS/PMS solution was added into wells of 96-well plate containing 100 μl culture medium. After culturing under 37 °C for 4 h, the absorbance at 490 nm was recorded by microplate reader.

### Real-time PCR

Total RNA was isolated using the RNA prep pure Cell/Bacteria Kit (Tiangen Biotech, Beijing, China), and reverse-transcription into complementary DNA was performed using the first complementary DNA Synthesis Kit with oligo (dT)15 (Tiangen Biotech). The levels of mRNA of genes of interest were measured by real-time PCR (7900 HT by Applied Biosystems, Foster City, CA, USA) using SYBR Green Master Mix (Roche Diagnostics, Indianapolis, IN, USA). Total amount of mRNA was normalized to endogenous β-actin mRNA. Sequences of PCR primer pairs were listed in Supplementary Table 1.

### Statistical analysis

Data are presented as mean ± SEM. Statistical significance was assessed by unpaired two-tailed Student’s *t*-test.

## Supplementary information

Supplementary Material

## Data Availability

RNA sequencing data that support the findings of this study have been deposited in GEO with the accession codes GSE117837
