# Supplementary material for: Single cell transcriptomic analysis of human mesenchymal stem cells reveals limited heterogeneity
Source: Cell Death Dis. 2019 May 8;10(5):368. doi: 10.1038/s41419-019-1583-4 (PMC6506509; doi:10.1038/s41419-019-1583-4)
Supplement: Supplementary file 1 — Supplementary Material [file 41419_2019_1583_MOESM1_ESM.pdf]

Figure S1

A

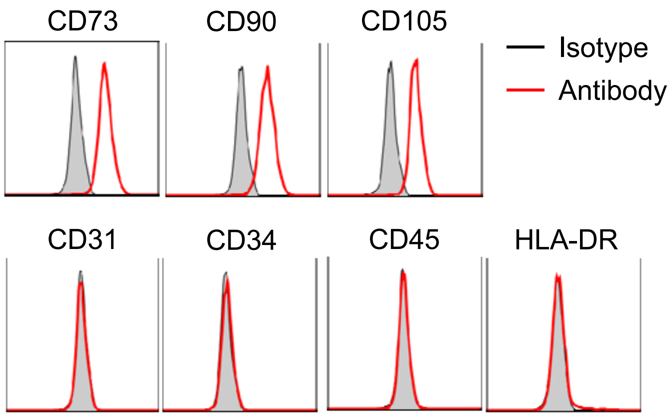

Figure S2

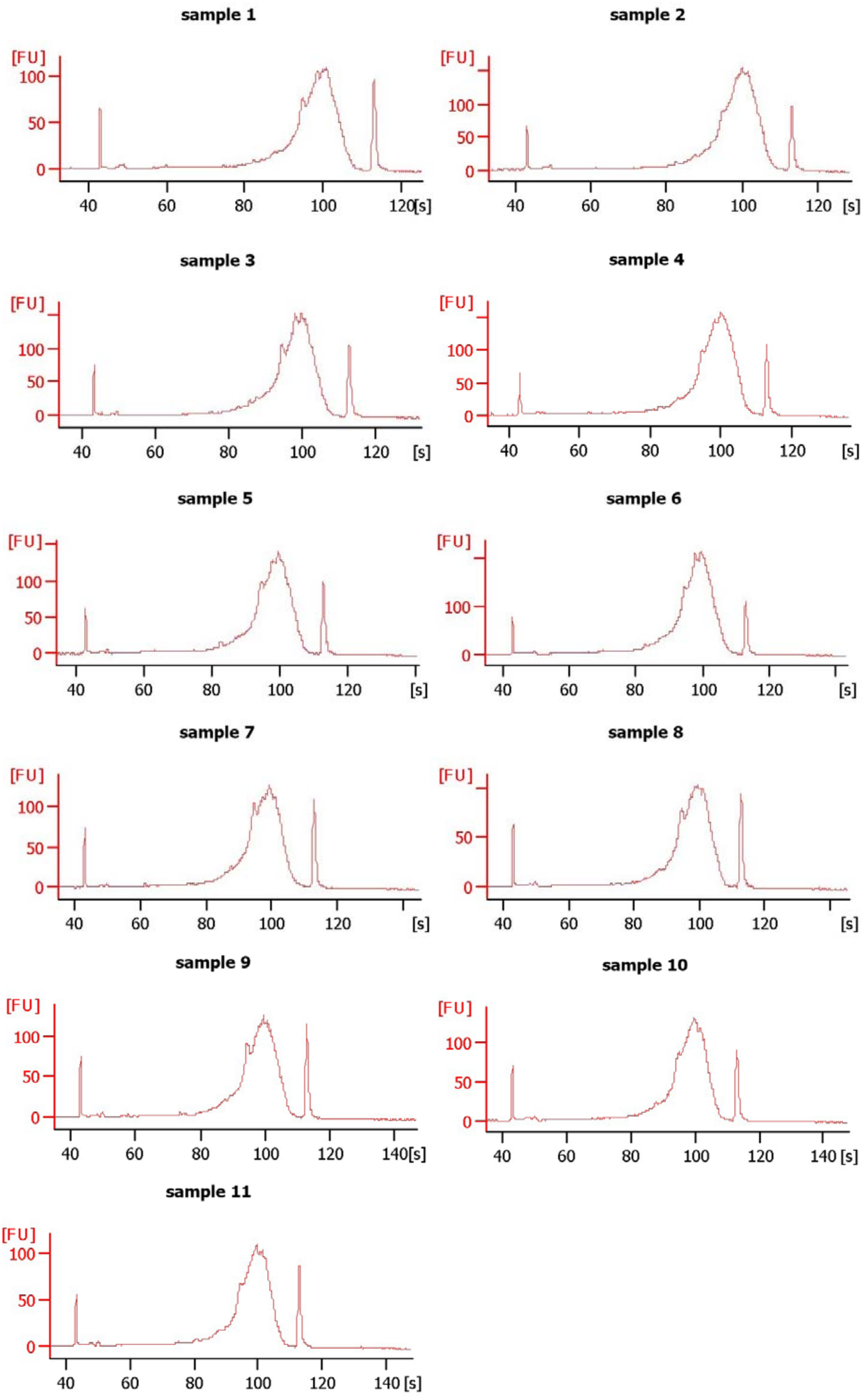

sample 1
[FU]
100
50
0
40 60 80 100 120 [s]
sample 2
[FU]
100
50
0
40 60 80 100 120 [s]
sample 3
[FU]
100
50
0
40 60 80 100 120 [s]
sample 4
[FU]
100
50
0
40 60 80 100 120 [s]
sample 5
[FU]
100
50
0
40 60 80 100 120 [s]
sample 6
[FU]
100
0
40 60 80 100 120 [s]
sample 7
[FU]
100
50
0
40 60 80 100 120 [s]
sample 8
[FU]
50
0
40 60 80 100 120 [s]
sample 9
[FU]
100
50
0
40 60 80 100 120 140 [s]
sample 10
[FU]
100
50
0
40 60 80 100 120 140 [s]
sample 11
[FU]
100
50
0
40 60 80 100 120 140 [s]

Figure S3

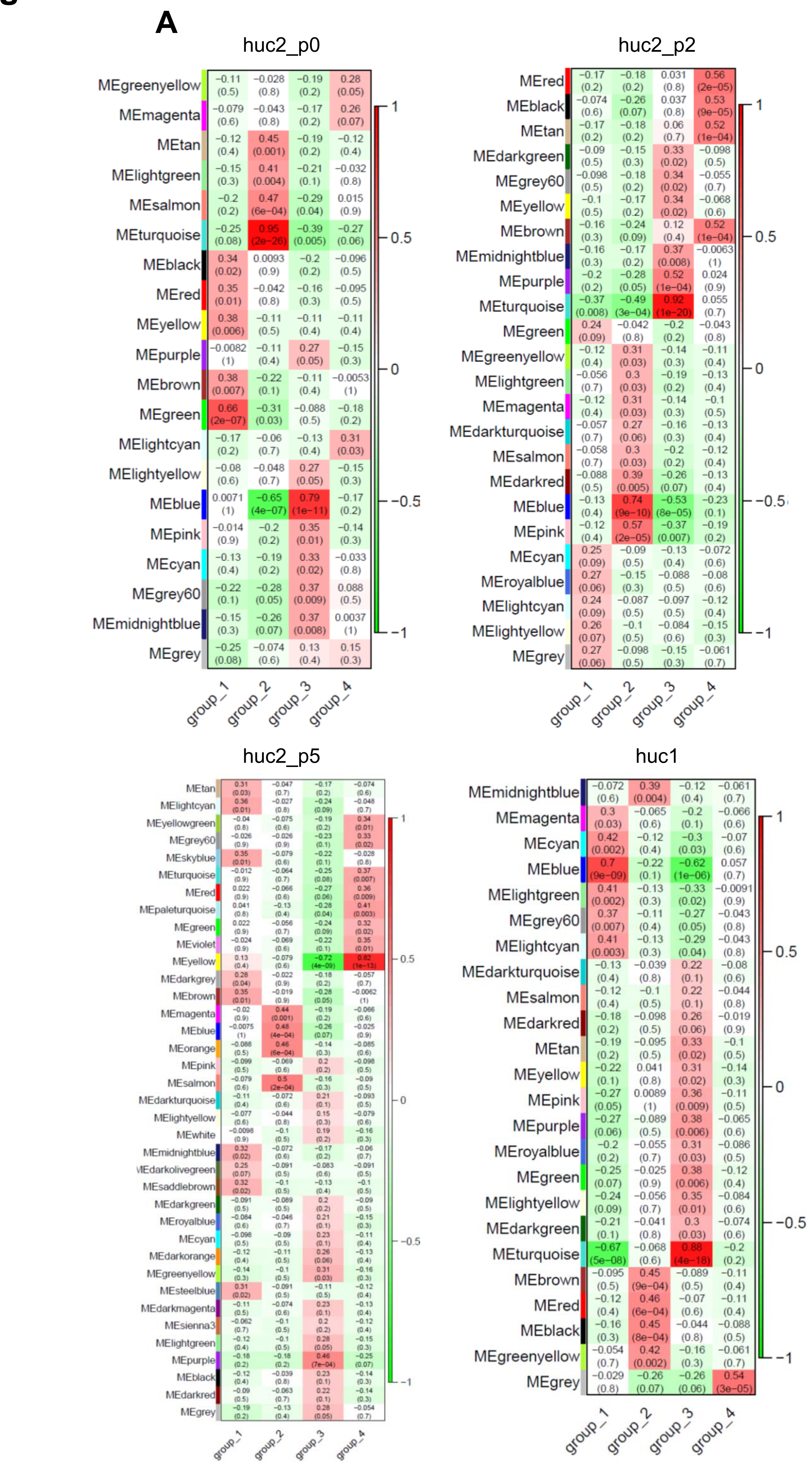

Figure S4

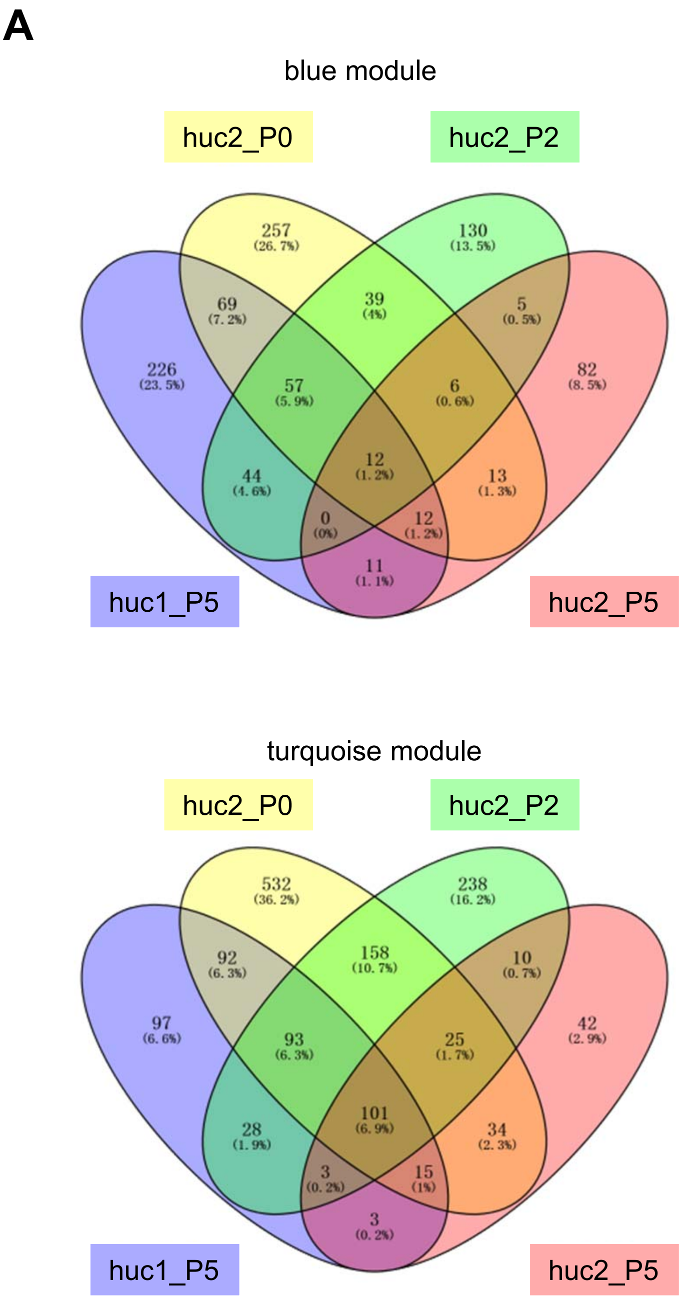
A
blue module
huc2_P0
huc2_P2
huc1_P5
huc2_P5
257
(26.7%)
130
(13.5%)
69
(7.2%)
39
(4%)
5
(0.5%)
226
(23.5%)
57
(5.9%)
6
(0.6%)
82
(8.5%)
12
(1.2%)
44
(4.6%)
13
(1.3%)
0
(0%)
12
(1.2%)
11
(1.1%)
turquoise module
huc2_P0
huc2_P2
huc1_P5
huc2_P5
532
(36.2%)
238
(16.2%)
92
(6.3%)
158
(10.7%)
10
(0.7%)
97
(6.6%)
93
(6.3%)
25
(1.7%)
42
(2.9%)
101
(6.9%)
28
(1.9%)
34
(2.3%)
3
(0.2%)
15
(1%)
3
(0.2%)

Figure S5

A

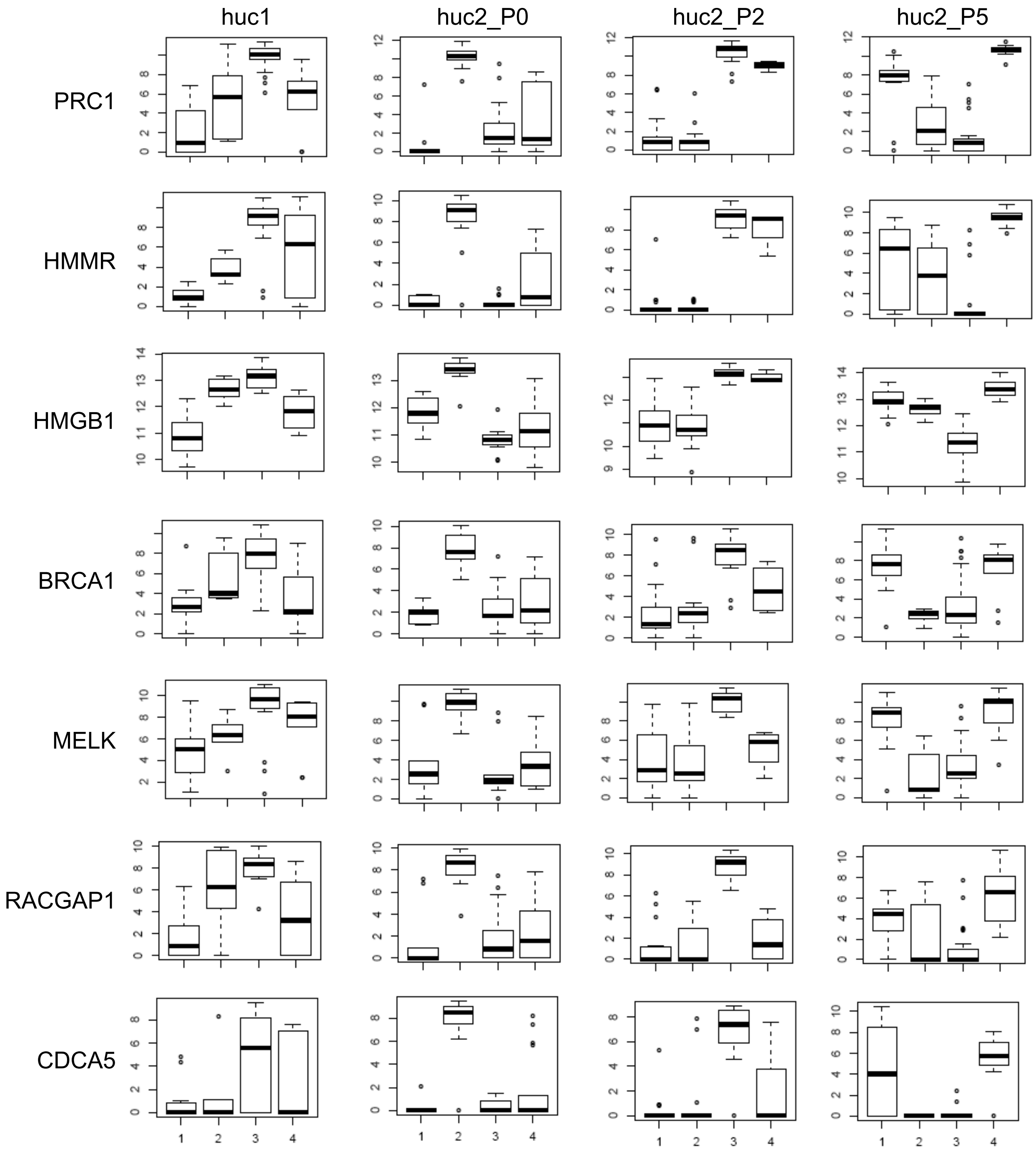

huc1
huc2_P0
huc2_P2
huc2_P5
PRC1
HMMR
HMGB1
BRCA1
MELK
RACGAP1
CDCA5
1
2
3
4

Figure S6

A

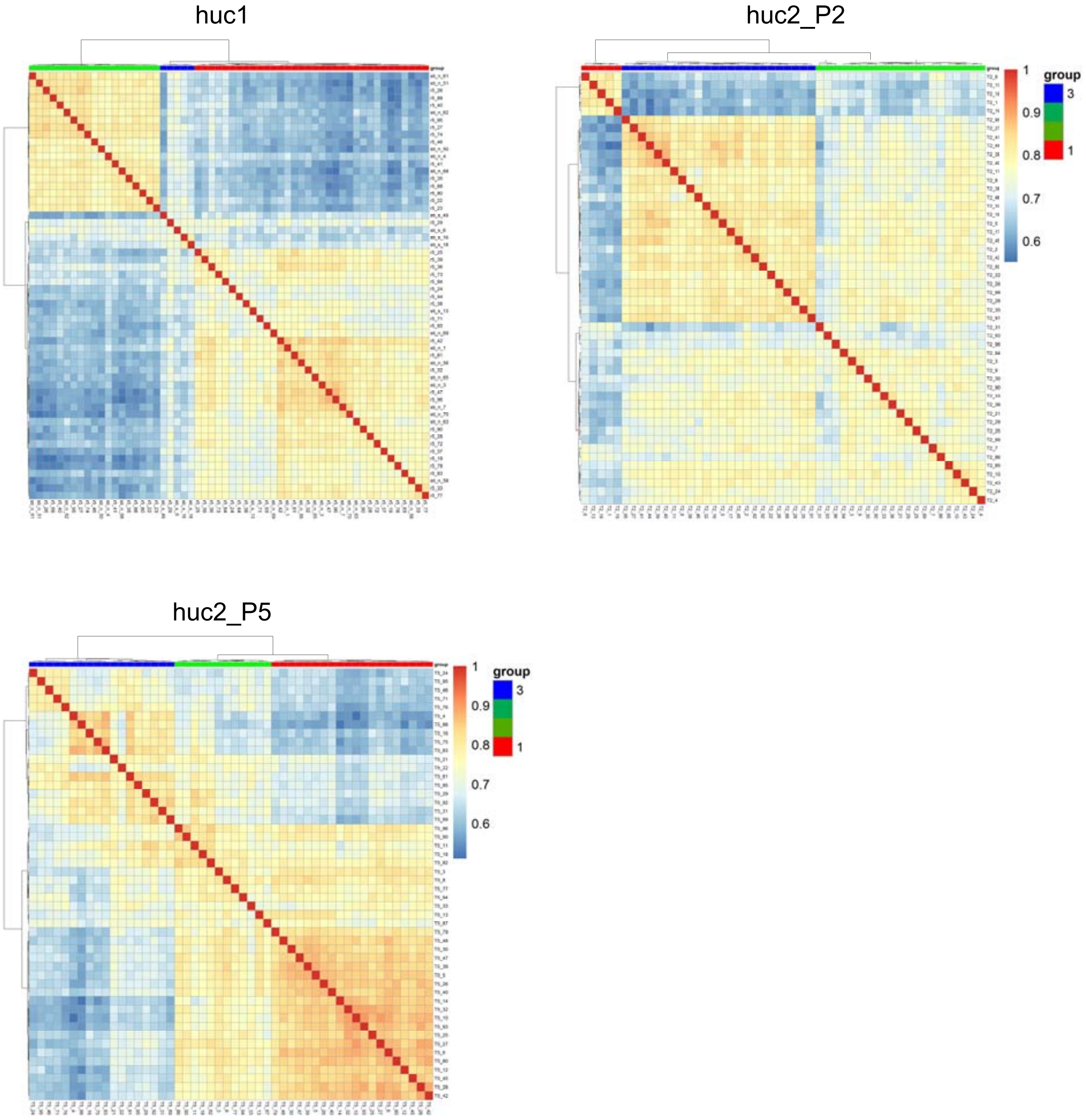

Figure S7

A

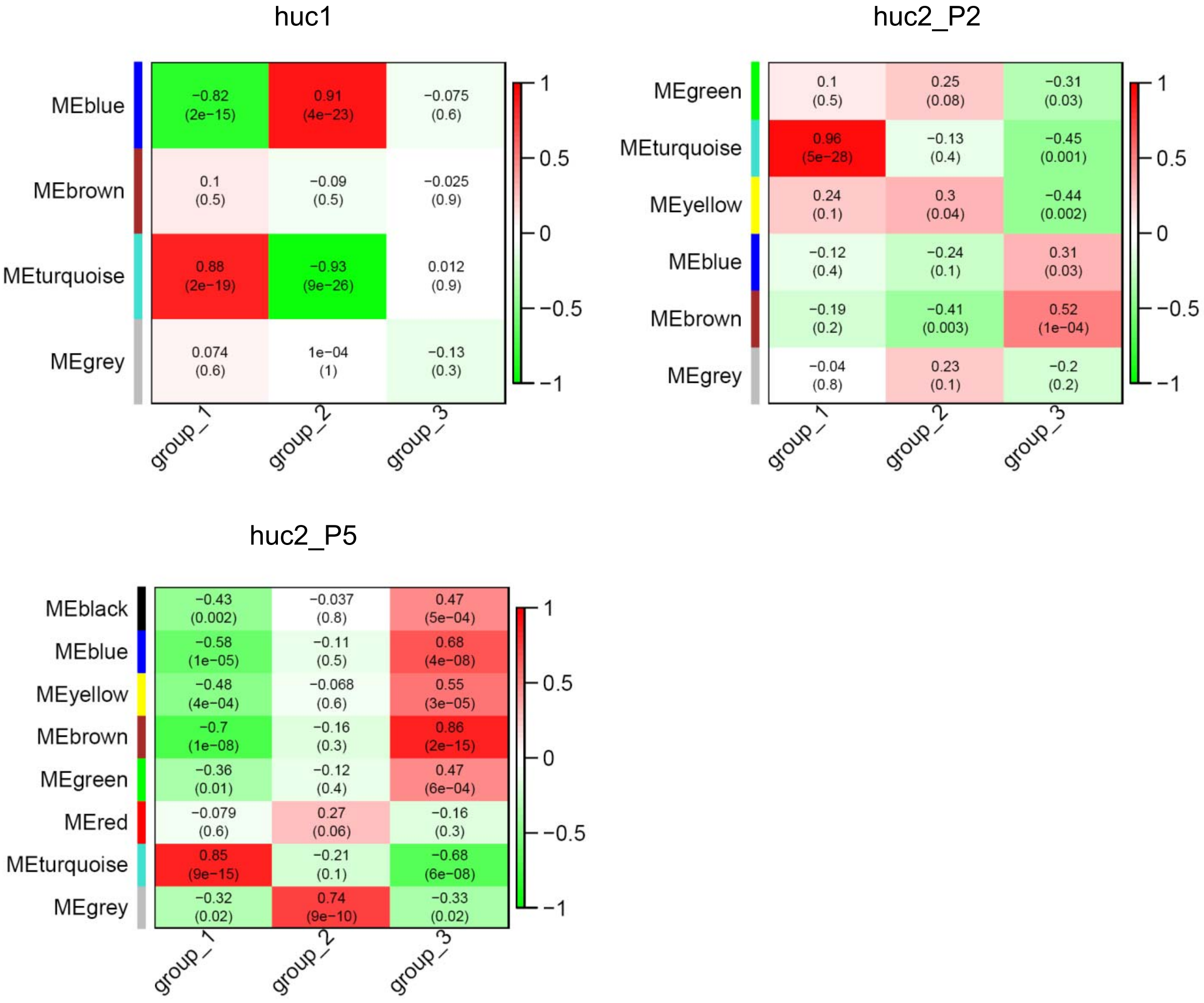
huc1
MEblue
MEbrown
MEturquoise
MEgrey
−0.82
(2e−15)
0.91
(4e−23)
−0.075
(0.6)
0.1
(0.5)
−0.09
(0.5)
−0.025
(0.9)
0.88
(2e−19)
−0.93
(9e−26)
0.012
(0.9)
0.074
(0.6)
1e−04
(1)
−0.13
(0.3)
group_1
group_2
group_3
1
0.5
0
−0.5
−1
huc2_P2
MEgreen
MEturquoise
MEyellow
MEblue
MEbrown
MEgrey
0.1
(0.5)
0.25
(0.08)
−0.31
(0.03)
0.96
(5e−28)
−0.13
(0.4)
−0.45
(0.001)
0.24
(0.1)
0.3
(0.04)
−0.44
(0.002)
−0.12
(0.4)
−0.24
(0.1)
0.31
(0.03)
−0.19
(0.2)
−0.41
(0.003)
0.52
(1e−04)
−0.04
(0.8)
0.23
(0.1)
−0.2
(0.2)
group_1
group_2
group_3
1
0.5
0
−0.5
−1
huc2_P5
MEblack
MEblue
MEyellow
MEbrown
MEgreen
MEred
MEturquoise
MEgrey
−0.43
(0.002)
−0.037
(0.8)
0.47
(5e−04)
−0.58
(1e−05)
−0.11
(0.5)
0.68
(4e−08)
−0.48
(4e−04)
−0.068
(0.6)
0.55
(3e−05)
−0.7
(1e−08)
−0.16
(0.3)
0.86
(2e−15)
−0.36
(0.01)
−0.12
(0.4)
0.47
(6e−04)
−0.079
(0.6)
0.27
(0.06)
−0.16
(0.3)
0.85
(9e−15)
−0.21
(0.1)
−0.68
(6e−08)
−0.32
(0.02)
0.74
(9e−10)
−0.33
(0.02)
group_1
group_2
group_3
1
0.5
0
−0.5
−1

Figure S8

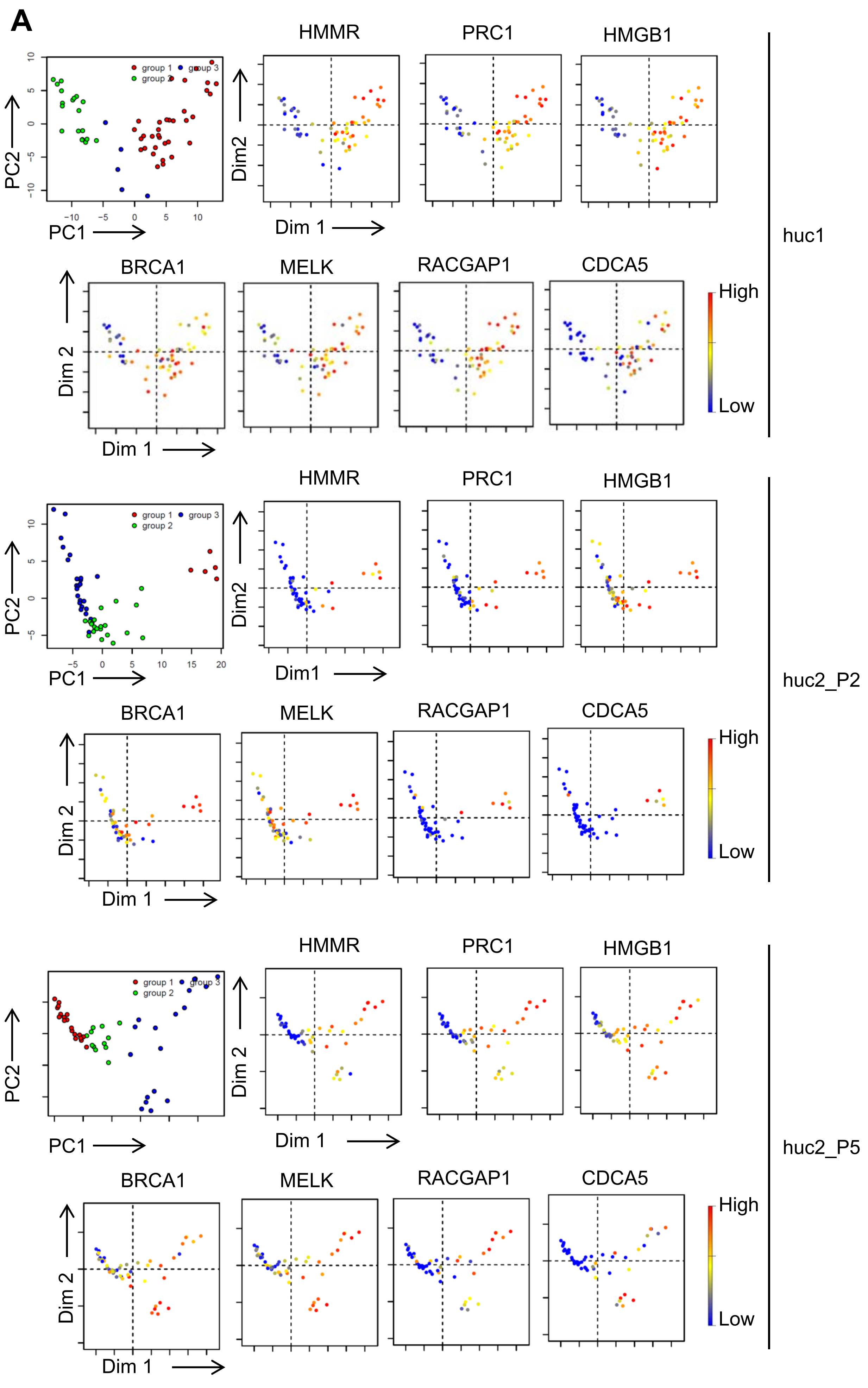
A
HMMR
PRC1
HMGB1
BRCA1
MELK
RACGAP1
CDCA5
PC1
PC2
Dim 1
Dim 2
Dim1
Dim2
group 1
group 2
group 3
High
Low
huc1
huc2_P2
huc2_P5

Figure S9

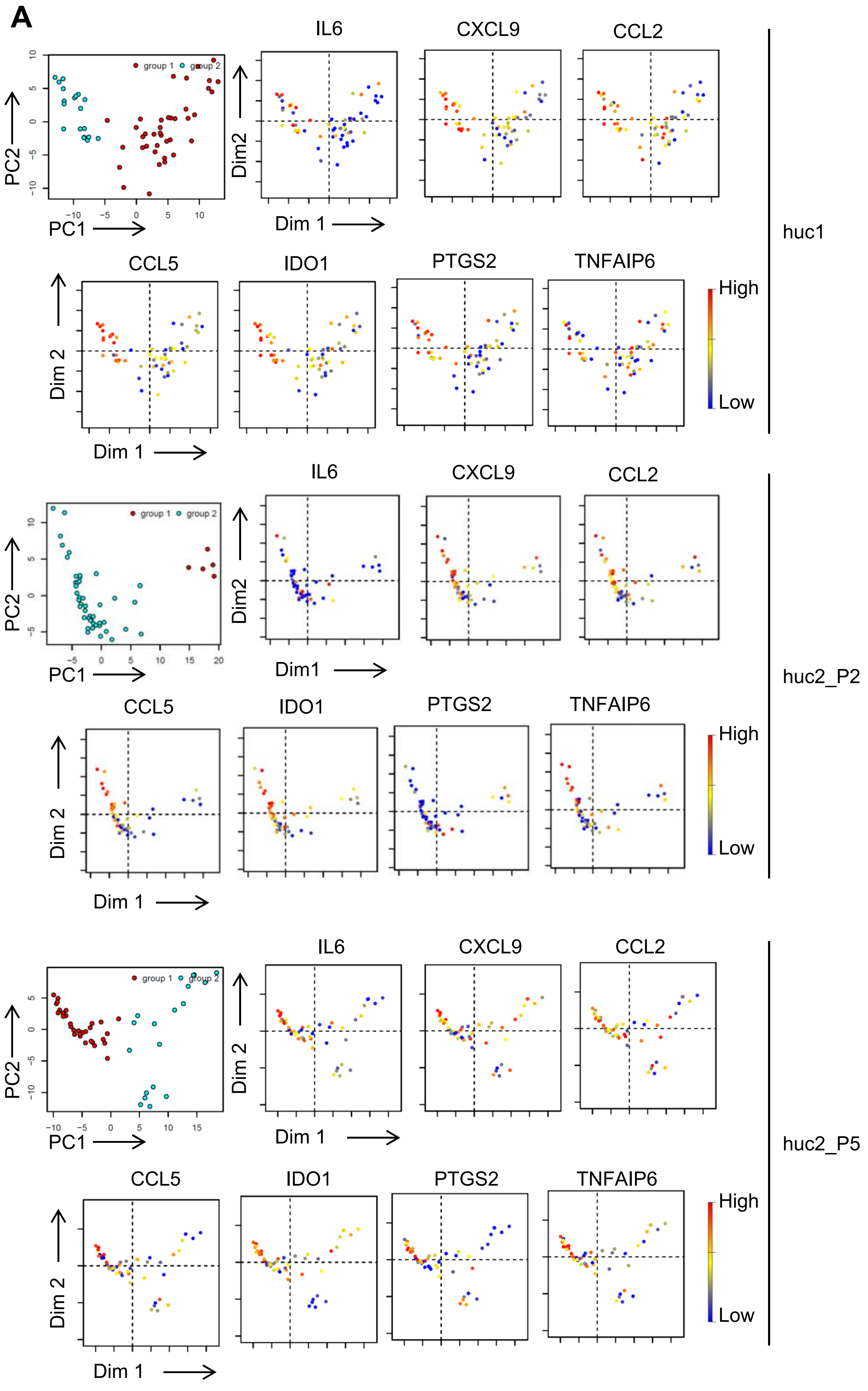
A
group 1
group 2
PC1
PC2
IL6
CXCL9
CCL2
CCL5
IDO1
PTGS2
TNFAIP6
Dim 1
Dim 2
Dim1
Dim2
High
Low
huc1
huc2_P2
huc2_P5

Figure S10

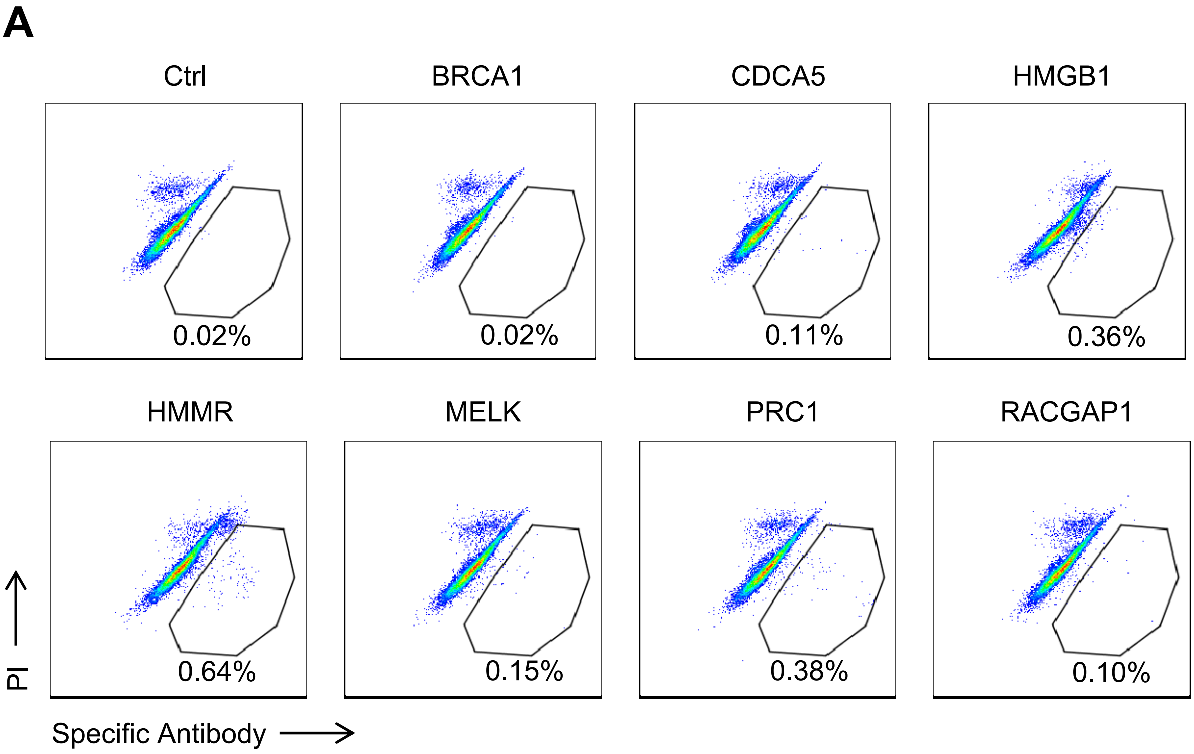
A
Ctrl
BRCA1
CDCA5
HMGB1
0.02%
0.02%
0.11%
0.36%
HMMR
MELK
PRC1
RACGAP1
0.64%
0.15%
0.38%
0.10%
PI
Specific Antibody

Figure S11

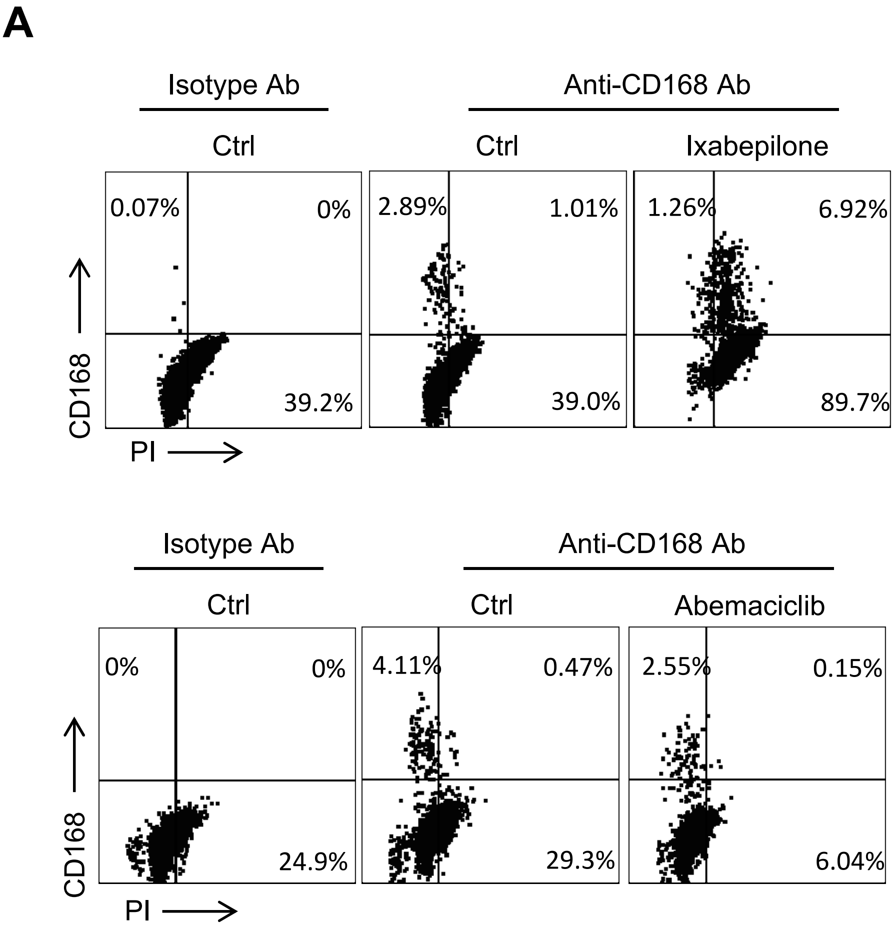
A
Isotype Ab
Anti-CD168 Ab
Ctrl
Ctrl
Ixabepilone
0.07%
0%
39.2%
2.89%
1.01%
39.0%
1.26%
6.92%
89.7%
CD168
PI
Isotype Ab
Anti-CD168 Ab
Ctrl
Ctrl
Abemaciclib
0%
0%
24.9%
4.11%
0.47%
29.3%
2.55%
0.15%
6.04%
CD168
PI

Supplementary Figure Legends

Fig S1.
The markers for MSC identification are analyzed by flow cytometry (gray shadow: isotype control, red line: marker of interest).

Fig S2.
Consistent cDNA quality is verified by Agilent 2100 bioanalyzer.

Fig S3.
WGCNA revealed key modules shown in heatmap, showing the significance of each module in each sub-cluster. Each cell contains the P value. Supplementary to Fig 1d.

Fig S4.
Venn plot showing the overlapped genes among four datasets in blue and turquoise modules. Supplementary to Fig 1e.

Fig S5.
Expression profile of featured genes shown as box plot. Supplementary to Fig 1f.

Fig S6.
Unsupervised hierarchical gene clustering of MSCs with IFNγ and TNFα pretreatment. Supplementary to Fig 2b.

Fig S7.
WGCNA revealed key modules shown in heatmap, showing the significance of each module in each sub-cluster. Each cell contains the p value. Supplementary to Fig 2c-2d.

Fig S8-9.
PCA analysis showing the clustering. Expression profile of featured genes (S7) and immune modulatory genes (S8), red to blue shows the level from high to low. Supplementary to Fig 2b-c.

Fig S10.
Specific antibody staining of featured genes on the surface of UC-MSCs.

Fig S11.
UC-MSCs were seeded to 80% consistency and treated with or without Ixabepilone (30nM) or Abemaciclib (4μM) for 36 hours. The DNA content and expression levels of CD168 were analyzed by flow cytometry after antibody and PI staining.

Supplementary Table 1 Primer sequence

ACTB-F	AGCCTCGCCTTTGCCGA
ACTB-R	GCGCGGCGATATCATCATC
PRC1-F	ATCACCTTCGGGAAATATGGGA
PRC1-R	TCTTTCTGACAGACGGATATGCT
HMGB1-F	TATGGCAAAAGCGGACAAGG
HMGB1-R	CTTCGCAACATCACCAATGGA
BRCA1-F	GAAACCGTGCCAAAAGACTTC
BRCA1-R	CCAAGGTTAGAGAGTTGGACAC
MELK-F	TCTCCCAGTAGCATTCTGCTT
MELK-R	TGATCCAGGGATGGTTCAATAGA
CDCA5-F	GAGGTCCCAGCGGAAATCAG
CDCA5-R	TCTTTAAGACGATGGGCTTTCTG
RACGAP1-F	ATGATGCTGAATGTGCGGAAT
RACGAP1-R	CGCCAACTGGATAAATTGGACTT
HMMR-F	ATGATGGCTAAGCAAGAAGGC
HMMR-R	TTTCCCTTGAGACTCTTCGAGA
MCM2-F	ATGGCGGAATCATCGGAATCC
MCM2-R	GGTGAGGGCATCAGTACGC
AURKB-F	CAGAAGAGCTGCACATTTGACG
AURKB-R	CCTTGAGCCCTAAGAGCAGATTT
CDK1-F	GGATGTGCTTATGCAGGATTCC
CDK1-R	CATGTACTGACCAGGAGGGATAG

Supplementary Table 2 Primer sequence for Fig 3H

hu-ANLN-F1	TGCCAGGCGAGAGAATCTTC
hu-ANLN-R1	CGCTTAGCATGAGTCATAGACCT
hu-ACTB-F	AGCCTCGCCTTTGCCGA
hu-ACTB-R	GCGCGGCGATATCATCATC
hu-Ang-1-F	TCGTGAGAGTACGACAGACCA
hu-Ang-1-R	TCTCCGACTTCATGTTTTCCAC
hu-ASF1B-F1	TCCGGTTCGAGATCAGCTTC
hu-ASF1B-R1	GTCGGCCTGAAAGACAAACA
hu-ASPM-F2	TGCAGTGGGTGAACATGAAAA
hu-ASPM-R2	CGAAGAGGGTGTTACCTCGTTT
hu-AURKA-F1	GAGGTCCAAAACGTGTTCTCG
hu-AURKA-R1	ACAGGATGAGGTACACTGGTTG
hu-AURKB-F3	CAGAAGAGCTGCACATTTGACG
hu-AURKB-R3	CCTTGAGCCCTAAGAGCAGATTT
hu-B7H4-F	CTTCTGCCTCTCAGCCCTTA
hu-B7H4-R	GAAATAGTTCTGTAGATCCCTGTTG
hu-BIRC5-F1	AGGACCACCGCATCTCTACAT
hu-BIRC5-R1	AAGTCTGGCTCGTTCTCAGTG
hu-BMP2-F	CCGACACTGAGACGCTGTTCCC
hu-BMP2-R	GACCTCGTCAGAGGGCTGGGAT
hu-BUB1B-F1	AAATGACCCTCTGGATGTTTGG
hu-BUB1B-R1	GCATAAACGCCCTAATTTAAGCC
hu-BUB1-F1	TGGGAAAGATACATACAGTGGGT
hu-BUB1-R1	AGGGGATGACAGGGTTCCAAT
hu-C11orf82-F1	AGGTTCAGATGCCAGTAACTTCT
hu-C11orf82-R1	AGTGATTGTTAGGTGCCTGAGA
hu-C12orf48-F2	TTGCTCAGGATTTGGATTTGAGG
hu-C12orf48-R2	TCAGTGGTGCTAAGAGCTACAA
hu-CASC5-F1	CTTCACACCGAGGACTCAAGA
hu-CASC5-R1	TTTGATGTGTAGAAGAGGCACTG
hu-CCDC34-F1	ACAGAAACAGGTGCGCTTACC
hu-CCDC34-R1	CAGCCGGTCACGTTCTTCTTT
hu-CCL2-F	GGCTGAGACTAACCCAGAAACA
hu-CCL2-R	GGAATGAAGGTGGCTGCTATGA
hu-CCL5-F	CTCCCCATATTCCTCGGACA
hu-CCL5-R	GTTGATGTACTCCCGAACCC
hu-CCNB1-F1	AATAAGGCGAAGATCAACATGGC
hu-CCNB1-F2	TTTGTTACCAATGTCCCCAAGAG
hu-CCNB2-F1	CCGACGGTGTCCAGTGATTT
hu-CCNB2-R1	TGTTGTTTTGGTGGGTTGAACT
hu-CCNE1-F	AAGGAGCGGGACACCATGA
hu-CCNE1-R	ACGGTCACGTTTGCCTTCC

hu-CCR10-F	CTGCTGGATACTGCCGATCT
hu-CCR10-R	GCGTAGAGAACGGGATTGAG
hu-CCR1-F	ATGGAAACTCCAAACACCAC
hu-CCR1-R	GGAGGTAGATGCTGGTCATG
hu-CCR2-F	TTCTGGAGACCTCAACCCAATG
hu-CCR2-R	GGCGGAGATACAGGGCAACTAA
hu-CCR3-F	TGACGCCTAAGCTATCACTGGA
hu-CCR3-R	CCGGATAAGCACAAGAAGACCA
hu-CCR4-F	AGAAGAAGAACAAGGCGGTGAA
hu-CCR4-R	CATGGTGGACTGCGTGTAAGAT
hu-CCR5-F	GTCCAATCTATGACATCA
hu-CCR5-R	GAATTGAGACTGACTGTA
hu-CCR8-F	TCCAGAACAAAGGCTGTCACTA
hu-CCR8-R	TTTGCACATTACAGTCCCAAAC
hu-CD105(ENG)-F	GGACGGTGACGGTGAAGGTGGA
hu-CD105(ENG)-R	TCTGGATCGGTGCGGGTGAGGT
hu-CD29(Intergrinb1)-F	TTCTGCACGATGTGATGATTTA
hu-CD29(Intergrinb1)-R	GTTCCTTTGCTACGGTTGGTTA
hu-CD31(PECAM-1)-F	GATCAGACGACCTCTAACGACC
hu-CD31(PECAM-1)-R	GCATAAGTAAGGCACCAGGAGTA
hu-CD34-F	CTACAACACCTAGTACCCTTGGA
hu-CD34-R	GGTGAACACTGTGCTGATTACA
hu-CD44-F	ACATCAGTCACAGACCTGCC
hu-CD44-R	GCAAACTGCAAGAATCAAAGC
hu-CD45(PTPRC)-F	GGAGAAGTTAGTAAAACCGAATC
hu-CD45(PTPRC)-R	TCTTATCAGACGAGGAACAATT
hu-CD73(NT5E)-F	GCCTGGGAGCTTACGATTTTG
hu-CD73(NT5E)-R	TAGTGCCCTGGTACTGGTCG
hu-CD90(THY1)-F	ATGAAGGTCCTCTACTTATCCGC
hu-CD90(THY1)-R	GCACTGTGACGTTCTGGGA
hu-CDC20-F	GCTTTGAACCTGAACGGTTTTG
hu-CDC20-R	TCTGGCGCATTTTGTGGTTTT
hu-CDC25C-F1	TCTACGGAACTCTTCTCATCCAC
hu-CDC25C-R1	TCCAGGAGCAGGTTTAACATTTT
hu-CDC45-F1	TTCGTGTCCGATTTCCGCAAA
hu-CDC45-R1	TGGAACCAGCGTATATTGCAC
hu-CDCA3-F1	AAGAGCGTCCCAGTCACAC
hu-CDCA3-R1	CCAGCACTAGGTGAACGGG
hu-CDCA5-F1	GAGGTCCCAGCGGAAATCAG
hu-CDCA5-R1	TCTTTAAGACGATGGGCTTTCTG
hu-CDCA8-F1	GAAGGGCAGTAGTCGGGTG
hu-CDCA8-R1	TCACGGTCGAAGTCTTTCAGA
hu-CDH1(E-Cad)-F	CCAAAGCCTCAGGTCATAAACA
hu-CDH1(E-Cad)-R	TTGGGTCGTTGTACTGAATGGT

hu-CDH2(N-Cad)-F	GACGGTTCGCCATCCAGAC
hu-CDH2(N-Cad)-R	TCGATTGGTTTGACCACGG
hu-CDK1-F	GGATGTGCTTATGCAGGATTCC
hu-CDK1-R	CATGTACTGACCAGGAGGGATAG
hu-CDKN3-F1	TCCGGGGCAATACAGACCAT
hu-CDKN3-R1	GCAGCTAATTTGTCCCGAAACTC
hu-CENPF-F1	CTCTCCCGTCAACAGCGTTC
hu-CENPF-R1	GTTGTGCATATTCTTGGCTTGC
hu-CENPK-F2	ATGGTACTGTCCACTAAGGAGTC
hu-CENPK-R2	TGTTCATCCAACCACCGTTGT
hu-CENPM-F1	GCGGACTCGATGCTCAAAGA
hu-CENPM-R1	TTCTGGAGACTGTATTTGCTGTG
hu-CEP55-F1	AGTAAGTGGGGATCGAAGCCT
hu-CEP55-R1	CTCAAGGACTCGAATTTTCTCCA
hu-CHAF1A-F1	AGCCCGTCTGCCGTTTAAG
hu-CHAR1A-R1	AGAAGTACCCTGATCGTCTGAC
hu-CKAP2L-F1	GAGCCAAAACACCAAGCCTTA
hu-CKAP2L-R1	GGAGTTTAATGCTGATGGACCTT
hu-CLSPN-F1	AAGACAGTGATTCCGAAACAGAG
hu-CLSPN-R1	TGCGCTTCAAGATTTTCCTGA
hu-COX2(PTGS2)-F	CTGGCGCTCAGCCATACAG
hu-COX2(PTGS2)-R	CGCACTTATACTGGTCAAATCCC
hu-CX3CR1-F	CAGCAAGAAGCCCAAGAGTGTC
hu-CX3CR1-R	GAAGAAGAAGGCGGTAGTGAAT
hu-CXCL10-F	GGCCATCAAGAATTTACTGAAAGCA
hu-CXCL10-R	TCTGTGTGGTCCATCCTTGGAA
hu-CXCL11-F	cttggctgtgatattgtgtgc
hu-CXCL11-R	gggtacattatggaggctttc
hu-CXCL12(SDF1)-F	ATGAACGCCAAGGTCG
hu-CXCL12(SDF1)-R	GGGCTACAATCTGAAGGG
hu-CXCL1-F	CCAAACCGAAGTCATAGCCACA
hu-CXCL1-R	CTTCTCCTAAGCGATGCTCAAA
hu-CXCL3-F	cactgttagggtaagggaatg
hu-CXCL3-R	caagggaaagagaaacgc
hu-CXCL9-F	AAGCAGCCAAGTCGGTTAGTGG
hu-CXCL9-R	GATAAGACGTTCGGGTGGGATC
hu-CXCR2-F	CCCAGTCAGGATTTAAGTTTACC
hu-CXCR2-R	GACCAGCATCACGAGGGAGTTT
hu-CXCR3-F	AGTGCTAAATGACGCCGAGGTTG
hu-CXCR3-R	GGGCTCCTGCGTAGAAGTTGATG
hu-CXCR4-F	ACTACACCGAGGAAATGGGCT
hu-CXCR4-R	CCCACAATGCCAGTTAAGAAGA
hu-CXCR5-F	GCTGCTCCGTGCTTGTTTGCTC
hu-CXCR5-R	TTGGCTGCTTGGCTTGAGTGGG

hu-DEPDC1-F1	TTTTGGTCCTGAAGTTACAAGGC
hu-DEPDC1-R1	TGGATACCTTCGTGGTAGAGTTT
hu-DKK1-F	GATCATAGCACCTTGGATGGG
hu-DKK1-R	GGCACAGTCTGATGACCGG
hu-DLGAP5-F2	TCCGACCTGGTCCAAGACAA
hu-DLGAP5-R2	GACGTGGGCATTACAGGCT
hu-DTL-F2	TCACTGGAATGCCGTCTTTGA
hu-DTL-R2	CTCACCAGCTTTTACGTCCC
hu-E2F1-F	ACGCTATGAGACCTCACTGAA
hu-E2F1-R	TCCTGGGTCAACCCCTCAAG
hu-EGF-F	TGGATGTGCTTGATAAGCGG
hu-EGF-R	ACCATGTCCTTTCCAGTGTGT
hu-EPO-F	AGGCCCTGTTGGTCAACTCT
hu-EPO-R	GCAGTGATTGTTCGGAGTGGA
hu-EREG-F	GGACAGTGCATCTATCTGGTGG
hu-EREG-R	TTGGTGGACGGTTAAAAAGAAGT
hu-ESCO2-F1	CACTGGGACGCACCCAAAA
hu-ESCO2-R1	CACTTGCCTTGTCGCAAAAG
hu-EXO1-F1	TGAGGAAGTATAAAGGGCAGGT
hu-EXO1-R1	AGTTTTTCAGCACAAGCAATAGC
hu-FAM64A-F1	CCTGGAAACGCCTGGAAAC
hu-FAM64A-R1	CAAAGCACTCTTAGCTGAGCG
hu-FANCB-F1	ATGAAGGATGGCCTAAGGGTC
hu-FANCB-R1	ACACACTAACAACTTTGCCAGT
hu-FANCD2-F1	AAAACGGGAGAGAGTCAGAATCA
hu-FANCD2-R1	ACGCTCACAAGACAAAAGGCA
hu-FANCI-F2	GACGAGCTATTGGATGTTGTCA
hu-FANCI-R2	TTCACGAGTTCTCTGCCTAGT
hu-FasL-F	TCCCCAGGATCTGGTGATG
hu-FasL-R	TCAGCACTGGTAAGATTGAACAC
hu-FGF2-F	CAAGCTACAACTTCAAGCAGAA
hu-FGF2-R	AAGAAACACTCATCCGTAACAC
hu-FOXM1-F1	CGTCGGCCACTGATTCTCAAA
hu-FOXM1-R1	GGCAGGGGATCTCTTAGGTTC
hu-Galectin-1 (LGALS1)-F	TCGCCAGCAACCTGAATCTC
hu-Galectin-1 (LGALS1)-R	GCACGAAGCTCTTAGCGTCA
hu-Galectin-3-F	GTGAAGCCCAATGCAAACAGA
hu-Galectin-3-R	AGCGTGGGTTAAAGTGGAAGG
hu-GAPDH-F	CTGGGCTACACTGAGCACC
hu-GAPDH-F1	CTGGGCTACACTGAGCACC
hu-GDNF-F	ACCTGGAGTTAATGTCCAACC
hu-GDNF-R	GGCATATTTGAGTCACTGCT
hu-GINS2-F1	CCCTGGTTTACCCGTGGAAG
hu-GINS2-R1	GGGAGCAGGCGACATTTCT

hu-GTSE1-F1	CAGGGGACGTGAACATGGATG
hu-GTSE1-R1	ATGTCCAAAGGGTCCGAAGAA
hu-HAS1-F	GAGCCTCTTCGCGTACCTG
hu-HAS1-R	CCTCCTGGTAGGCGGAGAT
hu-HGF-F	TCACGAGCATGACATGACTCC
hu-HGF-R	AGCTTACTTGCATCTGGTTCC
hu-HIST1H1B-F1	CTAAGGAGCGCAATGGCCTTT
hu-HIST1H1B-R1	CTTCGGAGTCTTCTTCACTGC
hu-HIST1H1E-F1	CTGGGGAAGCCAAGCCTAAG
hu-HIST1H1E-R1	CTGCTTTCGCCTTTTTCGGG
hu-HIST1H3C-F1	GCTTGCTACTAAAGCAGCCC
hu-HIST1H3C-R1	AGCGCACAGATTGGTGTCTTC
hu-HIST1H4C-F1	GCAAAGGCGGAAAAGGCTTG
hu-HIST1H4C-R1	TAGCCGGTTTTGTAATGCCCT
hu-HJURP-F1	CCACGCTGACCTACGAGAC
hu-HJURP-R1	CTCACCGCTTTTTGAATCGGC
hu-HLA-IG-F	CGCACAGACTGACAGAATGAACC
hu-HLA-IG-R	CGAGGTAATCCTTGCCATCGTAG
hu-HLA-II-F	TGGAAAGCACCTGTCTGTTG
hu-HLA-II-R	TGGTTGAGGTGCTGTGAGAG
hu-HMGB2-F1	CGGGGCAAAATGTCCTCGTA
hu-HMGB2-R1	CGGAAGAGTCCGGGTGTTT
hu-HMMR-F1	ATGATGGCTAAGCAAGAAGGC
hu-HMMR-R1	TTTCCCTTGAGACTCTTCGAGA
hu-HO-1-F	GGGCCAGCAACAAAGTGCAAGA
hu-HO-1-R	GAGTGTAAGGACCCATCGGAGAA
hu-Hsp90ab1(Mm.2180)-F	CATCTCCATGATTGGGCAGTT
hu-Hsp90ab1(Mm.2180)-R	CTTTGACCCGCCTCTCTTCTA
hu-ICAM1-F	gctatgccttgtcctcttg
hu-ICAM1-R	atacacacacacacacacgc
hu-IDO1-F	CGGTCTGGTGTATGAAGGGT
hu-IDO1-R	TGGCTTGCAGGAATCAGGAT
hu-IFNGR2-F1	CTCCTCAGCACCCGAAGATTC
hu-IFNGR2-R1	GCCGTGAACCATTTACTGTCG
hu-IGF1-F	GCTCTTCAGTTCGTGTGTGGA
hu-IGF1-R	GCCTCCTTAGATCACAGCTCC
hu-IGF2-F	GACTCGGCCTCTGGGAGGTTTA
hu-IGF2-R	ATTTGGCTCTTTGGGCTTCTGT
hu-IL10-F	TCAAGGCGCATGTGAACTCC
hu-IL10-R	GATGTCAAACTCACTCATGGCT
hu-IL11-F	CGAGCGGACCTACTGTCCTA
hu-IL11-R	GCCCAGTCAAGTGTCAGGTG
hu-IL1B-F	AGCTACGAATCTCCGACCAC
hu-IL1B-R	CGTTATCCCATGTGTCGAAGAA

hu-IL6-F	CACAGACAGCCACTCACCTC
hu-IL6-R	TTTTCTGCCAGTGCCTCTTT
hu-IL8 (CXCL8)-F	TCAGTGAAGATGCCAGTGAAAC
hu-IL8 (CXCL8)-R	ATCACATTCTAGCAAACCCATT
hu-INHBA-F	GGGGAGAACGGGTATGTGGAGA
hu-INHBA-R	AGAGGCGGATGGTGACTTTGGT
hu-iNOS-F	CCTGAGCTCTTCGAAATCCCA
hu-iNOS-R	CCCGAAACCACTCGTATTTGG
hu-IQGAP3-F1	GCAGCCTATGAACGCCTCA
hu-IQGAP3-R1	GGAGGGTGCAAAACAGTGG
hu-KGF(FGF7)-F	GGGGATATAAGAGTGAGAAGA
hu-KGF(FGF7)-R	ATAGGAAGAAAGTGGGCTGTT
hu-KIF14-F2	TGTAGGTAGATTGGCACTTCAGA
hu-KIF14-R2	CGACGTTGTAATGTAAGACGTGT
hu-KIF15-F2	AGGAATCTGTATTCGCAACTGTG
hu-KIF15-R2	ACTTCGTGGGATTACTCCTCTC
hu-KIF20B-F1	CATCCTTGGTCGGTTAAGTGAA
hu-KIF20B-R1	CCTTCTGTGTAGTTGCTGGGC
hu-KIF2C-F1	CTGTTTCCCGGTCTCGCTATC
hu-KIF2C-R1	AGAAGCTGTAAGAGTTCTGGGT
hu-KIF4A-F2	TGTAGGTAGATTGGCACTTCAGA
hu-KIF4A-R2	CGACGTTGTAATGTAAGACGTGT
hu-Klf4-F	TACTTACTCGCCTTGCTGATTGTCT
hu-Klf4-R	TGCTTGACGCAGTGTCTTCTCC
hu-LIF-F	GGCCCGGACACCCATAGACG
hu-LIF-R	CCACGCGCCATCCAGGTAAA
hu-MAD2L1-F2	GGACTCACCTTGCTTGTAACTAC
hu-MAD2L1-R2	GATCACTGAACGGATTTCATCCT
hu-MCM10-F2	CCCCTACAGACGATTTCTCGG
hu-MCM10-R2	CAGATGGGTTGAGTCGTTTCC
hu-MCM2-F1	ATGGCGGAATCATCGGAATCC
hu-MCM2-R1	GGTGAGGGCATCAGTACGC
hu-MCM5-F1	ATGTCGGGATTCGACGATCCT
hu-MCM5-R1	CCAGGTTGTAATGCCGCTTG
hu-MCM8-F1	AATGGAGAGTATAGAGGCAGAGG
hu-MCM8-R1	CAGAAGTACGTTTTCCTGTGGT
hu-MKI67-F1	ACGCCTGGTTACTATCAAAAGG
hu-MKI67-R1	CAGACCCATTTACTTGTGTTGGA
hu-MMP2-F	CAACTACGATGATGACCGCAAGT
hu-MMP2-R	GTCACAGTCCGCCAAATGAACC
hu-MMP9-F	TTCCAGTACCGAGAGAAAGCCTAT
hu-MMP9-R	GGTCACGTAGCCCACTTGGT
hu-MYBL2-F1	CCGGAGCAGAGGGATAGCA
hu-MYBL2-R1	CAGTGCGGTTAGGGAAGTGG

hu-Nanog-F	atgcctcacacggagactgt
hu-Nanog-R	aagtgggttgtttgcctttg
hu-NCAPG2-F1	TACAAGCCGTGTCTAAGGAGC
hu-NCAPG2-R1	TTGAGCCATGTTCGGTTTCCA
hu-NCAPG-F1	GAGGCTGCTGTCGATTAAGGA
hu-NCAPG-R1	AACTGTCTTATCATCCATCGTGC
hu-NCAPH-F2	AAACACGCAGATTACGGAACA
hu-NCAPH-R2	GTTGGTTGGTTCGGTGTCTTT
hu-NDC80-F1	CCTCTCCATGCAGGAGTTAAGA
hu-NDC80-R1	GGTCTCGGGTCCTTGATTTTCT
hu-NEIL3-F1	TCTCCTGTTTTGGAAGTGCAG
hu-NEIL3-R1	CATTAGCACATCACCTAGCATCC
hu-NEK2-F1	TGCTTCGTGAACTGAAACATCC
hu-NEK2-R1	CCAGAGTCAACTGAGTCATCACT
hu-nestin-F	ATAGAGGGCAAAGTGGTAAGCAG
hu-nestin-R	TTCTAGTGTCTCATGGCTCTGGTT
hu-netrin1-F	GACTTTGTCAATGCGGCCTTCGG
hu-netrin1-R	CGGGTTGTTGAGGTCGGTGAGG
hu-NUF2-F1	GGAAGGCTTCTTACCATTCAGC
hu-NUF2-R1	GACTTGTCCGTTTTGCTTTTGG
hu-NUSAP1-F1	AGCCCATCAATAAGGGAGGG
hu-NUSAP1-R1	ACCTGACACCCGTTTTAGCTG
hu-Oct4-F	GGGAGATTGATAACTGGTGTGTT
hu-Oct4-R	GTGTATATCCCAGGGTGATCCTC
hu-PAQR4-F2	GCTCTATCACCTCTTTATGTGCC
hu-PAQR4-R2	ACCCGACAACACAGTGTAGC
hu-PBK-F1	CCAAACATTGTTGGTTATCGTGC
hu-PBK-R1	GGCTGGCTTTATATCGTTCTTCT
hu-PDGFA-F	GCACTCGGTGAAGCAGTAAGAA
hu-PDGFA-R	TAAATACTACAGCGAGGAGGTG
hu-PD-L1(CD274)-F	GCACTGACATTCATCTTCCGTTTA
hu-PD-L1(CD274)-R	CTCCCTGTTTGACTCCATCTTT
hu-PD-L2-F	TGGACTCACCTCTGGAGCCTAT
hu-PD-L2-R	GAGTTTGTGCGAAGCCCTGTCT
hu-PLK1-F1	CACCAGCACGTCGTAGGATTC
hu-PLK1-R1	CCGTAGGTAGTATCGGGCCTC
hu-PLK4-F1	AAGCTCGACACTTCATGCACC
hu-PLK4-R1	GCATTTTCAGTTGAGTTGCCAG
hu-POLQ-F1	CTGCGTCGGAGTGGGAAAC
hu-POLQ-R1	CTGTAGGCTTGCATTCTCCTG
hu-PRC1-F1	ATCACCTTCGGGAAATATGGGA
hu-PRC1-R1	TCTTTCTGACAGACGGATATGCT
hu-PRR11-F2	AAAGATGGACCCATGCAGATAAC
hu-PRR11-R2	TGCTTTCGGCGATGGTATAAG

hu-P-selectin-F	CTACACCTGCTCCTGTTACCCT
hu-P-selectin-R	CCAACTAATGCAAATCCCTCTT
hu-RACGAP1-F1	ATGATGCTGAATGTGCGGAAT
hu-RACGAP1-R1	CGCCAACTGGATAAATTGGACTT
hu-RAD51AP1-F1	ATGACAAGCTCTACCAGAGAGAC
hu-RAD51AP1-R1	CACATTAGTGGTGACTGTTGGAA
hu-RANKL(TNFSF11)-F	CAACATATCGTTGGATCACAGCA
hu-RANKL(TNFSF11)-R	GACAGACTCACTTTATGGGAACC
hu-RFC3-F1	GTGGACAAGTATCGGCCCTG
hu-RFC3-R1	TGATGGTCCGTACACTAACAGAT
hu-RRM2-F1	CACGGAGCCGAAAACTAAAGC
hu-RRM2-R1	TCTGCCTTCTTATACATCTGCCA
hu-Semaphorin-3A(SEMA3A)-F	GTGCCAAGGCTGAAATTATCCT
hu-Semaphorin-3A(SEMA3A)-R	CCCACTTGCATTCATCTCTTCT
hu-SGOL1-F1	AACTCAGCAGTCACCTCATCT
hu-SGOL1-R1	TGCACCTACGTTTAGGCAGAG
hu-SHCBP1-F1	GCTACCGTGATAAACCAGGTTC
hu-SHCBP1-R1	AGGCTCTGAATCGCTCATAGA
hu-SKA1-F1	CCTGAACCCGTAAAGAAGCCT
hu-SKA1-R1	TCATGTACGAAGGAACACCATTG
hu-SKA3-F2	TGAGCGGTACATCGTATCCCA
hu-SKA3-R2	GGGGTTACAATTACGGGCTCT
hu-Sox2-F	ctccgggacatgatcagc
hu-Sox2-R	ggtagtgctgggacatgtgaa
hu-SPAG5-F2	CTGAGCAGTAGAACTGAGGCT
hu-SPAG5-R2	TCCACATGATTGACACGGAAAT
hu-SPC24-F1	GCCTTCCGCGACATAGAGG
hu-SPC24-R1	CCTGCTCCTTCGCATTGAGA
hu-SPC25-F1	ATGGTAGAGGACGAACTGGCA
hu-SPC25-R1	AGGAGGTGTCCGTACTTTTGAA
hu-ST3GAL2(SSEA4synthase)-F	AGGAGAATCCCTTGAACCGAGGAG
hu-ST3GAL2(SSEA4synthase)-R	CCATCATGCCCATTTCAGAACAAT
hu-TACC3-F1	TCGCCACCAGAAGTTACCG
hu-TACC3-R1	TCCCGCAGAGGTGTCTGAAA
hu-TGFa-F	AGGTCCGAAAACACTGTGAGT
hu-TGFa-R	AGCAAGCGGTTCTTCCCTTC
hu-TGFb-F	CAGCAACAATTCCTGGCGATACCT
hu-TGFb-R	AGCAGTGGGCGCTAAGGCGAAA

hu-TLR4-F	ATGCTAAGGTTGCCGCTTTCAC
hu-TLR4-R	CTCCCAGCTTTCTGGTCTCACG
hu-TMEM97-F1	TACCCAGTCGAGTTTAGAAACCT
hu-TMEM97-R1	TGTCATGGTGTGAACAGAGTAGA
hu-TOP2A-F1	ACCATTGCAGCCTGTAAATGA
hu-TOP2A-R1	GGGCGGAGCAAAATATGTTCC
hu-TP53-F	GCTGCTCAGATAGCGATGGTCT
hu-TP53-R	TTGTAGTGGATGGTGGTACAGTCAG
hu-TP63-F	ATTCAACGAGGGACAGATTGCC
hu-TP63-R	AAACTTGCTGCTTTCTGATGCTAT
hu-TP73-F	TACCTGTCCACCCTGAGTCCAGT
hu-TP73-R	ACCATCCGCCTTCAAGATTTATTT
hu-TPX2-F1	ATGGAACTGGAGGGCTTTTTC
hu-TPX2-R1	TGTTGTCAACTGGTTTCAAAGGT
hu-TRAIL(TNFSF10)-F	GCTCTGGGCCGCAAAAT
hu-TRAIL(TNFSF10)-R	TGCAAGTTGCTCAGGAATGAA
hu-TRIP13-F1	ACTGTTGCACTTCACATTTTCCA
hu-TRIP13-R1	TCGAGGAGATGGGATTTGACT
hu-TSG6-F	GGGCAGAGTTGGATACCCC
hu-TSG6-R	TGCGTGTGGGTTGTAGCAATA
hu-UBE2C-F1	GACCTGAGGTATAAGCTCTCGC
hu-UBE2C-R1	TTACCCTGGGTGTCCACGTT
hu-VCAM1-F	ACATGGAATTCGAACCCAAACA
hu-VCAM1-R	GGCTGACCAAGACGGTTGTATC
hu-VEGFA-F	ATGAACTTTCTGCTGTCTTGGGT
hu-VEGFA-R	TGGCCTTGGTGAGGTTTGATCC
hu-VEGFB-F	TAATGGGATTTGGGCTTTGGTA
hu-VEGFB-R	GCAGGAGGGCAGGCAGTCTGTA
hu-VEGFC-F	AGCACGAGCTACCTCAGCAAGA
hu-VEGFC-R	GCAGGGAACGTCTAATAATGGAA
hu-VEGI(TNFSF15)-F	ACCGAATGAACTATACCAACAA
hu-VEGI(TNFSF15)-R	GCTACCTACTTCGCATACAGAC
hu-WDHD1-F2	GCTTCAGGTCGTCCTAGACAG
hu-WDHD1-R2	CCTTTGGGATGTTACAAGTGGT
hu-ZWINT-F1	AGGACACTGCTAAGGGTCTCG
hu-ZWINT-R1	GCCTCTACGTGCTCCCTGTA
